# What plays beneath: mental health burden, personal resources, and treatment utilization among professional musicians and music students

**DOI:** 10.3389/fpsyg.2026.1924653

**Published:** 2026-08-21

**Authors:** Isabel Fernholz, Jens Plag, Ann Sophie Brehm, Stefanie Gestrich, Moritz Bruno Petzold, Alexander Schmidt, Antonia Bendau

**Affiliations:** 1 Berlin Center for Musicians Medicine (BCMM), Charité – Universitätsmedizin Berlin, Corporate Member of Freie Universität Berlin and Humboldt Universität zu Berlin, Berlin, Germany; 2 Department of Audiology and Phoniatrics CCM, Charité – Universitätsmedizin Berlin, Corporate Member of Freie Universität Berlin and Humboldt Universität zu Berlin, Berlin, Germany; 3 Department of Psychosomatic Medicine CCM, Charité – Universitätsmedizin Berlin, Corporate Member of Freie Universität Berlin and Humboldt Universität zu Berlin, Berlin, Germany; 4 Kurt Singer Institute for Music Physiology and Musicians’ Health, Hanns Eisler School of Music Berlin and University of the Arts Berlin, Berlin, Germany; 5 Faculty of Medicine, Institute for Mental Health and Behavioral Medicine, HMU Health and Medical University Potsdam, Potsdam, Germany; 6 Department of Psychiatry and Psychotherapy, Ernst von Bergmann Klinikum, Potsdam, Germany; 7 Oberberg Fachklinik Potsdam, Potsdam, Germany; 8 Department of Psychiatry and Neurosciences CCM, Charité – Universitätsmedizin Berlin, Corporate Member of Freie Universität Berlin and Humboldt Universität zu Berlin, Berlin, Germany; 9 Department of Psychology, MSB Medical School Berlin, Berlin, Germany; 10 Department of Psychology, Institute for Mental Health and Behavioral Medicine, HMU Health and Medical University, Potsdam, Germany

**Keywords:** anxiety, depression, distress, mental disorders, professional music, resilience, self-efficacy, self-esteem

## Abstract

**Introduction:**

Professional musicians may be exposed to distinctive occupational, psychosocial, and artistic demands, yet integrated evidence on their mental health, psychological resources, and treatment utilization remains scarce. Therefore, this study examined mental health indicators, including symptom burden and self-reported diagnosed mental disorders, personal psychological resources (resilience, self-efficacy, and self-esteem), occupational characteristics, and treatment use in a nationwide sample of professional musicians and music students in Germany.

**Methods:**

Data were collected through a large, anonymous, longitudinal online survey across different musical genres. Baseline data were obtained from 1,392 participants January–June 2024; 327 participants completed the follow-up in June 2025 (at least 1 year after baseline). Descriptive analyses, subgroup comparisons, cross-sectional regression models, and longitudinal regression models were used to examine symptom burden, diagnoses, resources, sociodemographic and occupational differences, and change over time.

**Results:**

At baseline, clinically relevant symptom levels were common: 30% of participants scored in the moderate or severe range for generalized anxiety symptoms, 32% scored in the moderate to severe range for depressive symptoms, and 22% reported a current diagnosed mental disorder. Employment status emerged as the most consistent subgroup correlate, with additional differences by gender, job role, and genre. Resilience, self-efficacy, and self-esteem were each negatively associated with depressive symptoms, generalized anxiety symptoms, and music performance anxiety, with self-esteem showing particularly strong associations. Overall, mental health indicators and personal resources were largely stable over 12 months, while music students reported higher depressive symptoms at follow-up beyond baseline levels. Treatment utilization was substantial, with half of participants reporting lifetime treatment for psychological complaints.

**Discussion:**

These findings indicate a substantial and unevenly distributed mental health burden among professional musicians in Germany. Although interpretation is limited by the observational design, non-probabilistic sampling, reliance on self-report, and substantial attrition at follow-up, the findings point to the relevance of prevention and support strategies that adequately address psychological resources and occupational conditions. Such provision may be broadly accessible while remaining responsive to differences in employment status, genre, gender-related experiences, barriers to care, support needs, and individual characteristics and contexts. These implications may guide future research and context-sensitive practice, although their effectiveness and clinical relevance require further evaluation

**Clinical trial registration:**

The study was pre-registered in the German Clinical Trials Register in January 2024 (https://drks.de/search/en/trial/DRKS00033401).

## 1 Introduction

The mental health of professional musicians has emerged as an area of growing clinical, societal, and scientific concern (Cardoso et al., 2025; Fernholz et al., 2025). While engagement with music is widely recognized as beneficial for psychological well-being in the general population (Viola et al., 2023), accumulating evidence suggests that pursuing music as a professional career may be associated with considerable mental health burden (Brehm et al., 2026; Cardoso et al., 2025; Kegelaers and Gibney, 2025; Willis et al., 2024; Berg et al., 2022). Professional music-making typically takes place within occupational contexts shaped by a complex interplay of artistic, psychosocial, and structural demands, whose nature, intensity, and relevance may vary across genres, career stages, employment arrangements, institutional settings, broader socio-economic conditions, and other factors (Détári et al., 2020; Kegelaers and Gibney, 2025; Rousseau et al., 2026; Willis et al., 2019). Existing research has, for example, repeatedly highlighted stressors related to performance pressure and public evaluation, employment insecurity, irregular and extended working hours, disruptions to private and social life, limited recovery, mandatory mobility and touring, and the physical demands of intensive practice and performance.

Consistent with these occupational stressors, a growing body of empirical research has documented elevated levels of symptom burden among musicians (Kenny et al., 2014; Vaag et al., 2016a; Brehm et al., 2026; Berg et al., 2022; Loveday et al., 2023). Within this literature, music performance anxiety has received by far the most sustained empirical attention, both as a symptom domain and as a target of intervention research (Fernholz et al., 2019; Nicholl and Abbott, 2025; Kinney et al., 2025). Other symptom domains and broader mental health-related aspects, including psychological resources, have been examined less systematically and have rarely been integrated within multi-domain frameworks (Cardoso et al., 2025; Kegelaers and Gibney, 2025). Consequently, the relative relevance, severity, and interrelations of different psychopathological symptoms, protective resources, and help-seeking variables remain insufficiently understood, as does their variation across musical genres, career stages, employment status, and other professional or sociodemographic characteristics.

A challenge for research in this field is that professional music does not constitute a homogeneous occupational context, but encompasses highly diverse working realities (Brehm et al., 2026; Musgrave et al., 2025; Cardoso et al., 2025). Mental health-related aspects in this field may therefore be shaped not only by individual vulnerability and protective factors, but also by broader contextual and occupational conditions. For example, some musicians work within relatively structured institutions; others navigate unstable freelance careers. Some are embedded in formal educational or orchestral systems, whereas others work in project-based, self-organized, or mixed professional settings. These differences may shape exposure to distinct occupational stressors, income security, institutional embeddedness, access to resources, social support, opportunities for mental health support, and other factors. Mental health patterns observed in one subgroup may therefore not generalize to the broader musician population.

To theoretically contextualize how heterogenous occupational conditions may influence mental health among professional musicians, broader frameworks on occupational health may offer useful conceptual perspectives. The Job Demands-Resources (JD-R) model, for example, provides a theoretical lens for examining how occupational demands and resources may jointly relate to mental health among professional musicians (Bakker and Demerouti, 2017; Xanthopoulou et al., 2007). It proposes that occupational demands require sustained physical or psychological effort and may particularly contribute to strain when they are persistent or insufficiently offset by occupational resources. Conservation of Resources theory further suggests that stress may arise when valued resources, including time, energy, financial security, social support, and perceived control, are threatened or depleted, or when substantial resource investment fails to produce an adequate return (Hobfoll et al., 2018). This may be particularly relevant in musical careers, where sustained personal and professional investments do not necessarily result in stable employment, recognition, or financial security (Berg et al., 2022). Repeated demands, particularly when accompanied by insufficient recovery or resource replenishment, may therefore contribute to cumulative resource loss, whereas access to personal, social, and occupational resources may support adaptation. A social-ecological perspective additionally emphasizes that mental health may be influenced by factors operating across multiple, potentially interacting levels, including individual, interpersonal, organizational, community, and broader societal or policy levels (McLeroy et al., 1988; Slimmen et al., 2024).

Empirical evidence capable of capturing aspects of this contextual complexity, however, remains limited. Much of the existing evidence remains based on relatively small and subgroup-specific samples, such as orchestral musicians, classical musicians, or conservatory students (Brehm et al., 2026; Kegelaers and Gibney, 2025; Cardoso et al., 2025; Willis et al., 2026). In addition, longitudinal data remain particularly scarce, with only a small number of studies available on longitudinal symptom progression in music students (Spahn et al., 2014; Rosset et al., 2022; Sadler and Miller, 2010). These limitations underscore the need for research that examines mental health among professional musicians across a broad range of symptom domains, psychological resources, and help-seeking variables, while also accounting for heterogeneity across musical, occupational, and sociodemographic subgroups.

Sex and gender constitute particularly relevant dimensions in this context—not only because sex and gender differences in mental health domains are well documented in the general population (Kayrouz et al., 2025; Ferrari et al., 2022; Christiansen et al., 2022), but also because many areas of professional music remain shaped by gendered occupational structures, including male-dominated networks, unequal access to opportunities, income disparities, and the underrepresentation of women and gender-diverse musicians in positions of visibility, leadership, production, and decision-making (Borgström Källén and Ferm Almqvist, 2025; Musicians’ Union, 2024).

Consistent with findings from the general population, where women often show higher rates of depression, anxiety disorders, and internalizing symptoms and men are more likely to show externalizing and substance-related problems (Kayrouz et al., 2025; Salk et al., 2017; McLean et al., 2011; Ferrari et al., 2022; Christiansen et al., 2022), several studies in musician samples have reported higher levels of music performance anxiety, social anxiety, trait anxiety, and depressive symptoms among female compared to male musicians (Kenny et al., 2014; Vaag et al., 2016a; Musgrave et al., 2025; Ackermann et al., 2014) and higher substance use among male and diverse musicians (Bendau et al., 2026). In addition, drawing on broader evidence on men’s mental health and help-seeking behavior (Üzümçeker, 2025), male musicians may be less likely to seek psychological support because of stigma or masculine role expectations, potentially contributing to under-detection or delayed help-seeking. More generally, sex- and gender-related patterns in musicians’ mental health remain insufficiently understood beyond selected symptom domains, particularly with regard to a broader range of mental health indicators, protective psychological resources, treatment utilization, and other mental health-related variables.

Consistent with the Job Demands-Resources model and Conservation of Resources theory, a resource-oriented perspective complements the examination of mental health burden by considering psychological resources that may support adaptation to occupational demands (Sisto et al., 2019; Wolke et al., 2025; Arbinaga, 2023). Building on emerging research on protective factors in musicians, a more systematic consideration of personal psychological resources may help understand how some individuals maintain psychological functioning despite occupational strain. Resilience, general self-efficacy, and self-esteem are three constructs with strong theoretical and empirical relevance for coping, adaptation, and psychological functioning and may therefore be particularly informative for prevention and targeted support (Schäfer et al., 2024; Singh et al., 2022; Sowislo and Orth, 2013).

Psychological resilience can be understood as the capacity or process of maintaining or regaining psychological functioning in the face of adversity or sustained stress (Troy et al., 2023; Sisto et al., 2019). Meta-analytic evidence indicates that resilience is negatively associated with psychopathological symptoms and positively associated with indicators of well-being across diverse non-musician populations (Hu et al., 2015). In musicians, resilience may be particularly relevant because musical careers often involve high work demands, intense competition, uncertainty, and recurring professional setbacks, requiring continuous adaptation to performance-related, occupational, and psychosocial stressors (Cardoso et al., 2025; Brehm et al., 2026; Kegelaers and Gibney, 2025). Initial (preliminary) studies support this assumption, showing that higher resilience is associated with fewer symptoms of depression and anxiety in classical musicians (Kegelaers et al., 2021) and linked to higher self-efficacy and lower maladaptive perfectionism among music students (Arbinaga, 2023). Constructs related to resilience, such as mental toughness, have also recently been discussed as potentially relevant individual-level resources in professional music contexts.^1^ Nevertheless, targeted evidence on resilience and related resources across the broader diversity of professional music contexts remains limited.

Self-efficacy represents another potentially important resource for coping with (occupational) stress (Schäfer et al., 2024). It can be conceptualized at different levels of specificity, ranging from general self-efficacy as a broad belief in one’s ability to manage difficult demands to domain-specific efficacy beliefs tied to particular tasks or contexts (Bandura, 1977; Schwarzer and Jerusalem, 1999). Music-related research has so far primarily examined domain-specific efficacy beliefs related to musical learning, practice, performance, and achievement: a recent meta-analysis, for example, found medium-sized positive associations between self-efficacy and musical achievement, as well as medium-sized negative associations between self-efficacy and music performance anxiety (Zelenak, 2024). These findings suggest that efficacy beliefs are relevant for musical functioning and may be amenable to intervention. However, because most studies have focused on musical or performance-related self-efficacy, general self-efficacy remains comparatively underexplored as a broader psychological resource in musicians’ mental health. This distinction is important because a musician may feel highly competent in performance-specific contexts while still perceiving limited capacity to manage career uncertainty, cope with professional setbacks, seek support, or navigate unstable working conditions.

Self-esteem represents a closely related, but conceptually distinct psychological resource: whereas self-efficacy concerns perceived capability, self-esteem reflects perceived personal worth, with global self-esteem referring to a person’s overall evaluation of their worth as an individual and domain-specific self-worth capturing perceived worth in specific areas of life or achievement (Rosenberg, 1965; Crocker and Wolfe, 2001). Self-esteem may be particularly relevant in professional music contexts, where careers are often shaped by repeated (critical) evaluation, comparison, feedback, and external validation (Willis et al., 2024). Under such conditions, global self-esteem may become closely intertwined with achievement, recognition, and perceived professional success (Crocker and Wolfe, 2001). Low self-esteem has been consistently associated with poorer mental health in the general population, including higher levels of depression and anxiety (Sowislo and Orth, 2013; Orth and Robins, 2014). In musician samples, lower self-esteem has also been linked to stronger depressive symptoms and music performance anxiety (Sickert et al., 2022). Beyond these initial findings, examining self-esteem together with resilience and general self-efficacy in larger and more diverse samples of professional musicians may provide a more comprehensive picture of psychological resources in these occupational contexts.

To contribute to a more differentiated understanding of musicians’ mental health, the present study examines mental health indicators, psychological resources, and subgroup differences in a large and heterogeneous sample of professional musicians and music students in Germany. Using data from a nationwide longitudinal online survey, we assessed key symptom domains, self-reported mental disorders, treatment utilization, and three psychological resources: resilience, general self-efficacy, and global self-esteem. Given the limited and fragmented evidence in this field, the study has a strong descriptive, exploratory, and integrative focus. We explored mental health indicators and psychological resources across musical genres, employment status, and gender groups, as well as self-reported mental disorders and treatment utilization. Based on the existing literature, we hypothesized that resilience (H1.1), general self-efficacy (H1.2), and global self-esteem (H1.3) would each be negatively associated with depressive symptoms (a), generalized anxiety (b), and music performance anxiety (c). We further expected women and gender-diverse musicians to report higher levels of depressive symptoms (H2a), generalized anxiety symptoms (H2b), and music performance anxiety (H2c) than men. Moreover, we hypothesized that symptom burden would differ by employment status, with music students and freelance musicians reporting higher levels of depressive symptoms (H3a), generalized anxiety symptoms (H3b), and music performance anxiety (H3c) than employed musicians.

By integrating symptom burden and diagnoses, psychological resources, treatment utilization, and subgroup comparisons, this study aims to provide an empirical basis for more targeted prevention, counseling, and intervention approaches in professional music contexts.

## 2 Materials and methods

### 2.1 Study design and procedure

The present study was conducted within a large research project of the Berlin Center for Musicians’ Medicine (BCMM) and the Department of Psychiatry and Neurosciences CCM at Charité – Universitätsmedizin Berlin. It used a nationwide anonymous online survey design to examine mental health, psychological resources, and profession-related characteristics among professional musicians and music students in Germany. The study comprised a large baseline assessment between January and June 2024 (T1), followed by a second assessment in June 2025 (T2; at least 1 year after baseline participation) in a smaller longitudinal subsample. Both survey waves were administered online using SoSci Survey (Leiner, 2019). The study was approved by the Ethics Committee of Charité – Universitätsmedizin Berlin (EA1/327/21) and was preregistered in the German Clinical Trials Register (DRKS00033401) before the start of baseline data collection.

The target population consisted of adult professional musicians and music students across Germany, reflecting the study’s aim to capture mental health across different stages of musical training and professional development. It included musicians from different musical genres, instrumental or vocal groups, and employment contexts. Sufficient proficiency in German or English was required to understand the study information and complete the questionnaire. Participation was voluntary and not compensated. Before entering the survey, participants received written information about the study aims, procedures, data protection, and their right to withdraw from the study or skip individual questions at any time. Informed consent was obtained electronically before access to the survey was granted.

Participants were recruited using a broad nationwide convenience sampling strategy designed to reach a large and heterogeneous sample across musical genres, career stages, and employment contexts. Recruitment took place via email distribution lists, newsletters, and social media channels of music-related institutions, professional associations, and initiatives. For example, all music universities and higher education institutions with music-related degree programs in Germany were contacted to reach music students, while professional musicians were particularly approached through organizations and networks such as unisono, Initiative Musik, Musicboard Berlin, and other music-related associations.

Participants who indicated willingness to take part in the follow-up assessment provided an email address for recontact at baseline. To preserve anonymity, these email addresses were stored separately from survey data and processed independently of the main dataset. Of the 585 participants who provided contact information and were invited to the follow-up via e-mail, 327 (55.9%) took part in the second assessment. Baseline and follow-up responses were linked using non-identifying participant codes.

### 2.2 Assessment

#### 2.2.1 Survey structure and scope

Baseline and follow-up assessments included largely the same set of self-report measures to allow comparability between measurement points. Some detailed sociodemographic and profession-related variables, such as information on musical training and career onset, were assessed only at baseline. At both waves, participants provided information on key sociodemographic characteristics and profession-related variables, including musical genre, instrument or voice group, and employment status. The overarching survey included additional modules addressing further aspects of musicians’ mental health and professional experiences^2^; the following section describes only the measures relevant to the present analyses. Unless stated otherwise, scale scores were computed according to the respective instrument manuals or validation studies, with higher scores indicating higher levels of the construct assessed. Internal consistencies in the present sample are reported as Cronbach’s *α* and presented in Table 1.

**Table 1:** Descriptive statistics, reliability, and T1-T2 change by scale.

Time point			Descriptive statistics						Reliability		Change from T1 to T2		
	*n*	*M*	SD	Min	Q1	Median	Q3	Max	Cronbach’s *α* [95% CI]	Δ (T2 − T1)	*t*	*p*	Cohen’s *d*
Depressive symptoms													
T1	1,306	7.94	5.45	0	4	7	11.0	27	0.863 [0.852, 0.873]	−0.05	−0.20	0.843	−0.011
T2	313	7.69	5.16	0	4	7	10.0	24	0.848 [0.837, 0.860]	—	—	—	—
Generalized anxiety symptoms													
T1	1,297	7.53	4.89	0	4	7	10.0	21	0.882 [0.873, 0.891]	0.04	0.16	0.870	0.009
T2	308	7.30	4.92	0	3	6	10.0	21	0.886 [0.877, 0.895]	—	—	—	—
Music performance anxiety													
T1	1,158	51.22	14.71	20	40	50	61.0	90	0.927 [0.922, 0.933]	−1.26	−2.46	0.015	−0.146
T2	291	49.65	14.86	20	38	48	59.5	94	0.931 [0.926, 0.936]	—	—	—	—
Resilience													
T1	1,105	60.17	8.99	23	55	61	67.0	77	0.836 [0.823, 0.849]	0.29	0.73	0.469	0.042
T2	295	60.97	8.72	30	56	62	67.0	77	0.828 [0.814, 0.841]	—	—	—	—
Self-efficacy													
T1	1,136	28.35	4.76	10	26	29	31.0	40	0.869 [0.858, 0.879]	0.34	1.62	0.107	0.096
T2	289	28.92	4.67	15	26	29	31.0	40	0.878 [0.869, 0.888]	—	—	—	—
Self-esteem													
T1	1,150	3.15	1.01	1	2	3	4.0	5	—	0.03	0.64	0.524	0.037
T2	299	3.17	0.97	1	3	3	4.0	5	—	—	—	—	—
Psychological health status													
T1	1,367	6.61	2.12	0	5	7	8.0	10	—	0.10	0.93	0.3553	0.052
T2	315	6.74	2.00	0	5	7	8.0	10	—	—	—	—	—
Physical health status													
T1	1,365	6.81	1.86	0	6	7	8.0	10	—	0.11	1.11	0.2671	0.063
T2	315	7.01	1.78	2	6	7	8.0	10	—	—	—	—	—

Δ, *t*, *p*, and Cohen’s *d* refer to the paired-sample comparison (participants with valid data at both T1 and T2).

#### 2.2.2 Mental health indicators

Music performance anxiety was assessed using the Bühnenangstfragebogen (BAF; Fehm et al., 2002), a German adaptation of the Performance Anxiety Questionnaire (Cox and Kenardy, 1993). It comprises 20 items assessing the extent of symptoms of music performance anxiety, rated on a five-point scale ranging from “never” to “always.” Two subscale scores reflecting cognitive and somatic anxiety symptoms, as well as a total score, can be calculated.

Generalized anxiety symptoms were assessed using the Generalized Anxiety Disorder Scale-7 (GAD-7; Spitzer et al., 2006). The GAD-7 consists of seven items referring to anxiety symptoms during the past 2 weeks. Items are rated from 0 (“not at all”) to 3 (“nearly every day”), yielding a total score between 0 and 21. Scores of 5, 10, and 15 are commonly interpreted as cut-offs for mild, moderate, and severe anxiety symptoms, respectively.

Depressive symptoms were examined using the Patient Health Questionnaire-9 (PHQ-9; Kroenke et al., 2001). The PHQ-9 comprises nine items assessing depressive symptoms during the past 2 weeks. Items are rated on a four-point scale from 0 (“not at all”) to 3 (“nearly every day”), resulting in a total score ranging from 0 to 27. Established cut-offs are commonly used to indicate minimal (0–4), mild (5–9), moderate (10–14), moderately severe (15–19), and severe (20–27) depressive symptom levels.

Mental disorders were assessed using two binary items asking participants whether they currently had one or more diagnosed mental disorders (point prevalence) and whether they had had one or more diagnosed mental disorders during the past 12 months (12-month prevalence). If endorsed, participants were asked to specify the disorder(s) in a free-text field (subsequently coded manually according to ICD-10 categories) and to indicate the source of diagnosis: self-diagnosis, psychiatrist or psychotherapist, general practitioner, or other.

In addition, participants rated their current mental and physical health status using two single-item numeric rating scales. Participants indicated their current mental and physical health status separately on a scale from 0 (“worst imaginable”) to 10 (“best imaginable”). These items were used as brief subjective indicators of perceived mental and physical health.

#### 2.2.3 Treatment utilization

At baseline, lifetime treatment history was assessed with a binary item asking participants whether they had ever received psychiatric or psychotherapeutic treatment. Current treatment was assessed by asking whether participants were currently receiving psychotherapy, medication, or other forms of treatment, with multiple responses possible. At follow-up, treatment utilization during the past 12 months was assessed with the same structure to capture treatment use between baseline and follow-up.

#### 2.2.4 Psychological resources

Resilience was assessed using the 11-item Resilience Scale (RS-11; Schumacher et al., 2005), a German short version of the Resilience Scale originally developed by Wagnild and Young (1993). The RS-11 measures resilience as a personal resource reflecting adaptive coping and psychological resilience in the face of stress. Items are rated on a seven-point Likert scale ranging from 1 (“strongly disagree”) to 7 (“strongly agree”). Total scores range from 11 to 77, with higher scores indicating greater resilience.

General self-efficacy was assessed using the General Self-Efficacy Scale (GSE; Schwarzer and Jerusalem, 1999). The GSE comprises 10 items assessing a broad belief in one’s ability to cope with difficult demands and successfully manage challenging situations. Items are rated on a four-point scale from 1 (“not at all true”) to 4 (“exactly true”), resulting in total scores ranging 10 from to 40. Higher scores indicate stronger general self-efficacy beliefs.

Global self-esteem was assessed using the Single-Item Self-Esteem Scale (SISE; Robins et al., 2001), with the item “I have high self-esteem” rated on a 5-point scale ranging from “not at all true” to “very true.”

### 2.3 Data analysis

All analyses were conducted in R (Version 4.6.0; R Core Team, 2026), using the car, lmtest, and sandwich packages for robust inference and the psych package for reliability and descriptive statistics. To reduce the risk of survey dropout and inattentive or insincere responses, item responses were not mandatory. Consequently, the number of valid observations varied across variables and analyses. All analyses were conducted using all available valid cases for the respective variables. Reflecting the study’s broad and integrative scope, the analytic strategy included a strong descriptive component, comprising summary statistics, subgroup comparisons, and graphical displays to characterize the sample and examine patterns across key study variables. Regarding inferential analyses, a two-tailed alpha level of 0.05 was used for all significance tests.

Pearson correlations were computed among the six main constructs at T1 to assess their bivariate associations. Following Cohen’s (1988) guidelines, correlation effect sizes were interpreted as small (*r* ≥ 0.10), moderate (*r* ≥ 0.30), or large (*r* ≥ 0.50). Group differences in T1 scores across the five covariates were screened using Kruskal-Wallis tests because of right-skewed raw distributions for several constructs (skewness −0.63 to 0.92).

Cross-sectional associations between the main study variables (depressive symptoms, generalized anxiety symptoms, music performance anxiety, resilience, self-efficacy, and self-esteem) at T1 were examined with linear regression, controlling for the covariates gender, employment status, and genre. Each variable was entered individually and centered. A combined model entering all three resources simultaneously was used to control for intercorrelations.

Longitudinal associations were examined with three linear regression models predicting each T2 symptom burden outcome (depressive symptoms, generalized anxiety symptoms, music performance anxiety) from its own centered T1 baseline, plus gender, employment status, and genre. This approach tests whether the covariates are associated with T2 levels beyond what is already explained by the T1 baseline. A fourth model predicted T2 psychological health from centered T1 physical health, T1 psychological health as a second predictor, and the same three covariates.

Model assumptions were checked for every regression model: heteroskedasticity (Breusch-Pagan test), normality of residuals (Shapiro–Wilk test), skewness and kurtosis of residuals, and influential cases (Cook’s distance). Skewness of residuals ranged from −0.76 to 0.99 and kurtosis from −0.13 to 2.39, both well within commonly applied thresholds for problematic non-normality, and the maximum Cook’s distance across all models was 0.13, far below the conventional threshold of 1.0. Breusch-Pagan tests indicated heteroskedasticity in most models, therefore heteroskedasticity-consistent robust standard errors were used for all models.

## 3 Results

### 3.1 Sociodemographic characteristics

The full T1 sample comprised *N* = 1,392 participants; *n* = 327 also completed the T2 assessment. At T1, the sample had a mean age of 39.3 years (SD = 14.7). A larger share of participants was female (58.7%; male: 39.7%; diverse/other: 1.6%) and classically trained (70.8%; popular: 29.2%). Just over half were employed (52.0%; freelance: 18.9%; students: 27.2%), and most held an academic degree (71.1%). Participants who completed T1 only did not differ significantly from those who also participated at T2 in age, gender, employment status, job role, or instrument group (all *p* ≥ 0.15), nor in baseline symptom severity (depressive symptoms, generalized anxiety symptoms, music performance anxiety; all *p* ≥ 0.13), but classical musicians were significantly more likely to remain in the study through T2 than popular musicians (*χ*^2^ (1) = 9.44, *p* = 0.002), indicating that attrition was selective with respect to genre but not with respect to the other demographic, occupational, or clinical characteristics assessed. All sociodemographic and occupational characteristics of the baseline sample (T1) and the longitudinal follow-up sample (T2) are reported in Table 2.

**Table 2:** Sociodemographic and occupational characteristics of the sample at T1 and T2.

Variable	Baseline T1 (*N* = 1,392)	Follow-up T2 (*N* = 327)
Age (*M* ± SD)	39.3 ± 14.7	39.7 ± 14.1
Gender		
Female	810 (58.7%)	200 (61.5%)
Male	547 (39.7%)	116 (35.7%)
Diverse/Other	22 (1.6%)	9 (2.8%)
Employment status		
Employed	724 (52.0%)	175 (53.5%)
Freelance	263 (18.9%)	68 (20.8%)
Student	379 (27.2%)	80 (24.5%)
Other^a^	26 (1.9%)	4 (1.2%)
Education		
Secondary education or lower	47 (3.4%)	8 (2.5%)
Academic entrance qualification	350 (25.4%)	72 (22.2%)
Academic degree	979 (71.1%)	244 (75.3%)
Genre		
Classical	985 (70.8%)	254 (77.7%)
Popular^b^	407 (29.2%)	73 (22.3%)
Job role		
Orchestra tutti	412 (29.6%)	104 (31.8%)
Choir tutti	129 (9.3%)	34 (10.4%)
Orchestra solo	241 (17.3%)	57 (17.4%)
Choir solo	57 (4.1%)	18 (5.5%)
Instrumental/voice solo	122 (8.8%)	27 (8.3%)
Ensemble	78 (5.6%)	14 (4.3%)
Music teaching	265 (19.0%)	59 (18%)
Other	79 (5.7%)	14 (4.3%)
No response	9 (0.6%)	0 (0.0%)
Instrument group		
Strings (bowed)	392 (28.2%)	92 (28.1%)
Winds (woodwinds)	201 (14.4%)	51 (15.6%)
Brass	127 (9.1%)	31 (9.5%)
Percussion	57 (4.1%)	9 (2.8%)
Singing	222 (15.9%)	59 (18.0%)
Keyboard	142 (10.2%)	38 (11.6%)
Plucked strings	42 (3.0%)	8 (2.4%)
Multiple instruments	146 (10.5%)	33 (10.1%)
Other	17 (1.2%)	2 (0.6%)
No response	46 (3.3%)	4 (1.2%)

Values are presented as *n* (%) unless otherwise indicated. Percentages are based on available responses and may therefore not correspond to the total sample size in each column.

a The “other” employment status category was excluded from all subsequent analyses involving employment status because of its small size and heterogeneous composition.

b For analytic feasibility, the popular music category was combined across the following genres, in descending order of frequency: pop, jazz/blues, rock/metal/punk, other genres, electronic music, folk/country, and hip-hop/rap.

### 3.2 Descriptive statistics, reliability, and change over time

Descriptive statistics, reliability coefficients, and within-person change from T1 to T2 for the six main constructs (depressive symptoms, generalized anxiety symptoms, and music performance anxiety; resilience, self-efficacy, and self-esteem) and for the additional constructs (physical health status and mental health status) are presented in Table 1. Change scores reflect the individual-level difference between each participant’s T1 and T2 value, restricted to participants with non-missing data at both time points, and were tested with paired-sample *t*-tests; Cohen’s *d* was computed from the mean and standard deviation of these individual-level differences. At the group level, scores remained mostly stable; after Bonferroni correction, none of the examined constructs showed a statistically significant change between T1 and T2.

Applying established clinical cutoffs, 31.5% of participants scored at or above the screening threshold for clinically relevant depressive symptoms (PHQ-9 ≥ 10; Kroenke et al., 2001) at T1, with a severity distribution of minimal 30.3%, mild 38.1%, moderate 18.5%, moderately severe 8.7%, and severe 4.4%. For generalized anxiety symptoms, 29.9% of participants scored at or above the screening threshold (GAD-7 ≥ 10; Spitzer et al., 2006) at T1, with a severity distribution of minimal 29.8%, mild 40.3%, moderate 19.0%, and severe 10.9%. Both measures showed little change at T2 (PHQ-9 ≥ 10: 29.1%; GAD-7 ≥ 10: 30.2%).

### 3.3 Correlations among main constructs

Pearson correlations among the six main constructs at T1 are presented in Table 3. Following Cohen’s (1988) guidelines, depressive and generalized anxiety symptoms were strongly correlated (*r* = 0.77). The three personal-resource measures were moderately to strongly intercorrelated (*r* = 0.48 to 0.57) and each was moderately negatively correlated with all three symptom measures (*r* = −0.27 to −0.49).

**Table 3:** Correlation matrix of mental health indicators and personal resources at T1.

Scale	Depressive symptoms	Generalized anxiety symptoms	Music performance anxiety	Self-efficacy	Self-esteem
Depressive symptoms	—				
Generalized anxiety symptoms	0.77	—			
Music performance anxiety	0.48	0.51	—		
Self-efficacy	−0.38	−0.35	−0.41	—	
Self-esteem	−0.45	−0.40	−0.49	0.57	—
Resilience	−0.46	−0.38	−0.27	0.54	0.48

Pearson correlations were calculated using pairwise complete observations.

### 3.4 Gender, genre, employment status, professional role, and instrument

**Figure 1:**
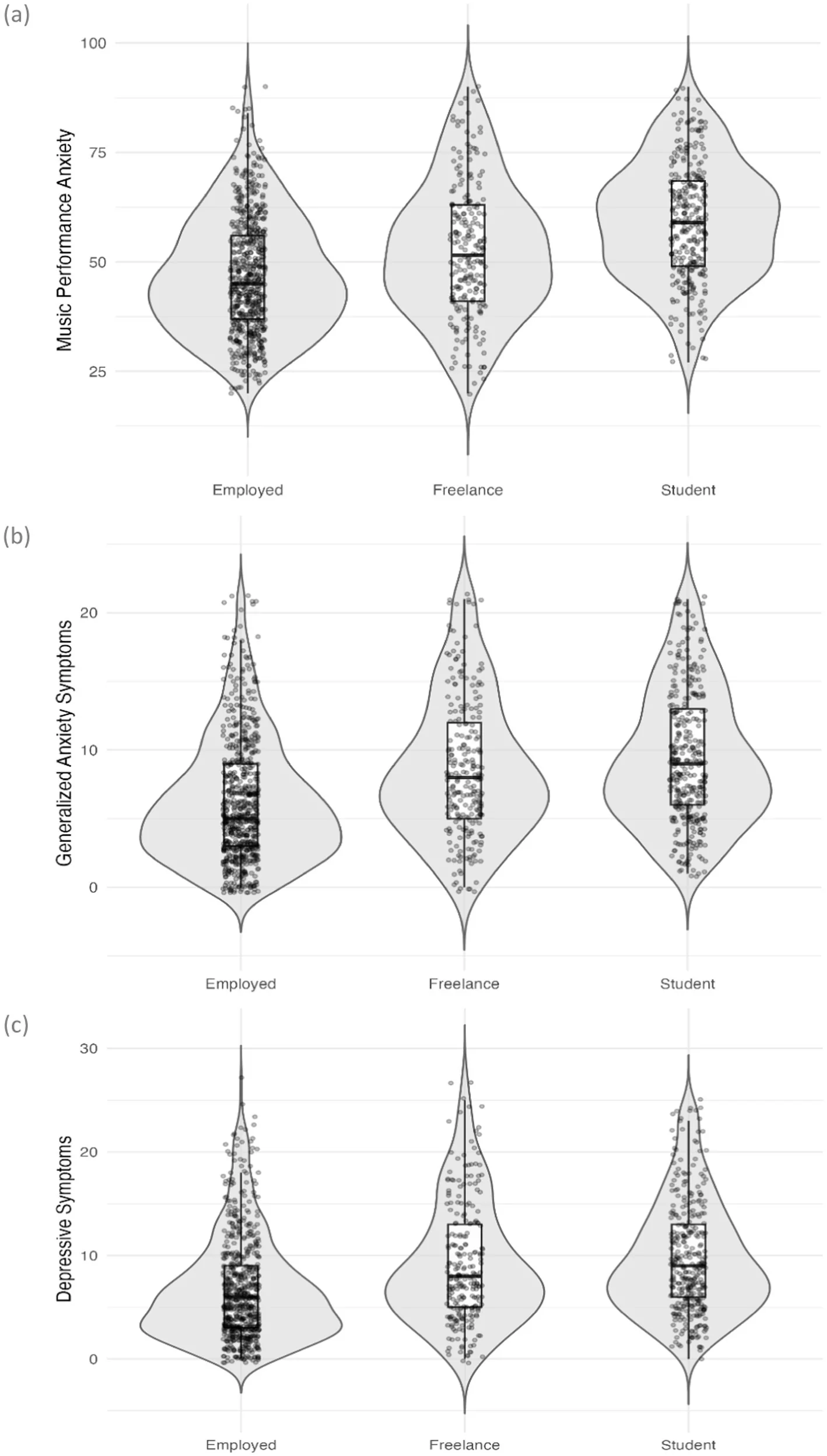
Distributions of **(a)** music performance anxiety, **(b)** generalized anxiety symptoms, and **(c)** depressive symptoms by employment status at T1. Boxes show the median and interquartile range; jittered points represent individual scores.

**Figure 2:**
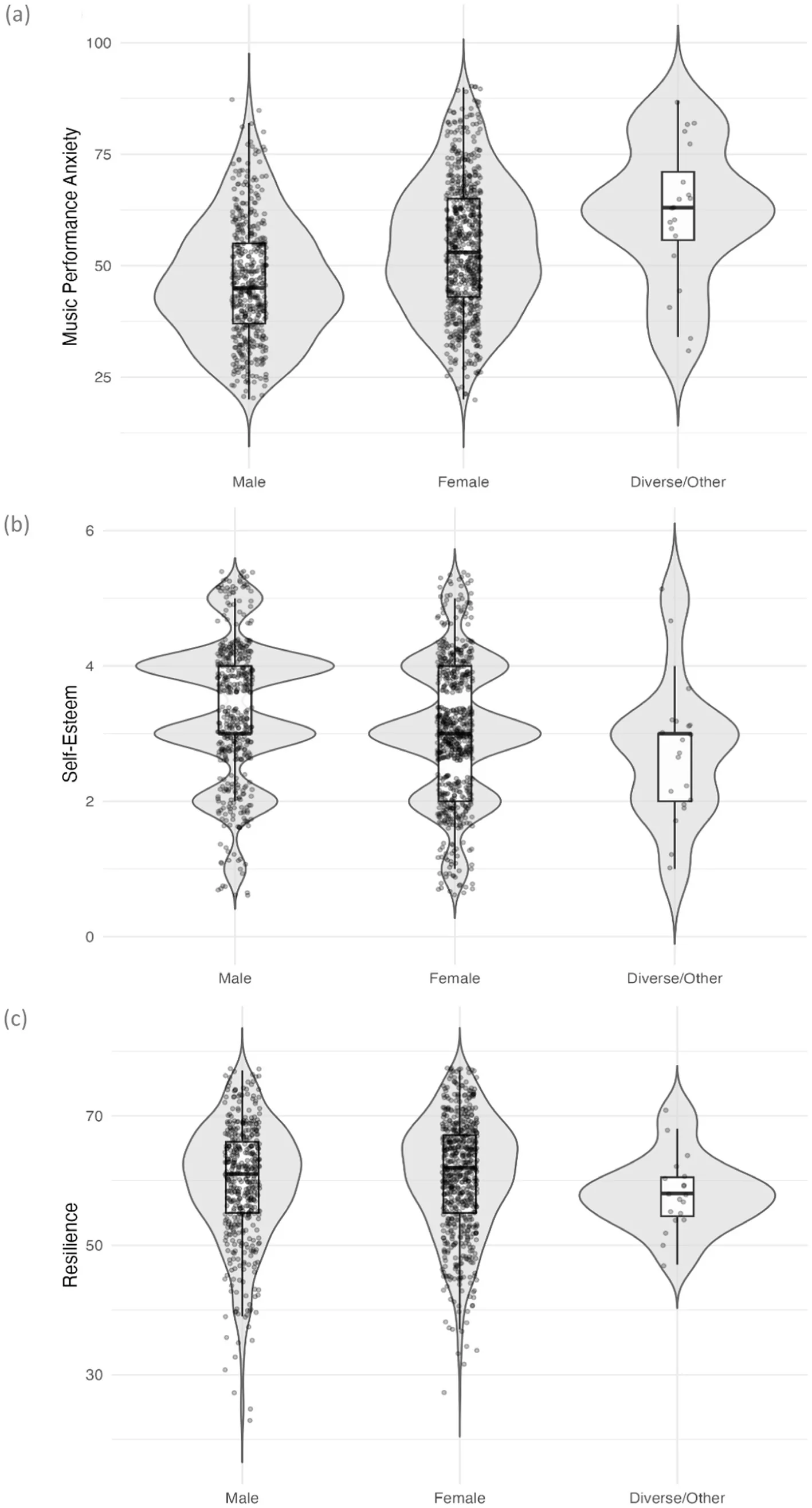
Distributions of **(a)** music performance anxiety, **(b)** self-esteem, and **(c)** resilience by gender at T1. Boxes show the median and interquartile range; jittered points represent individual scores.

**Figure 3:**
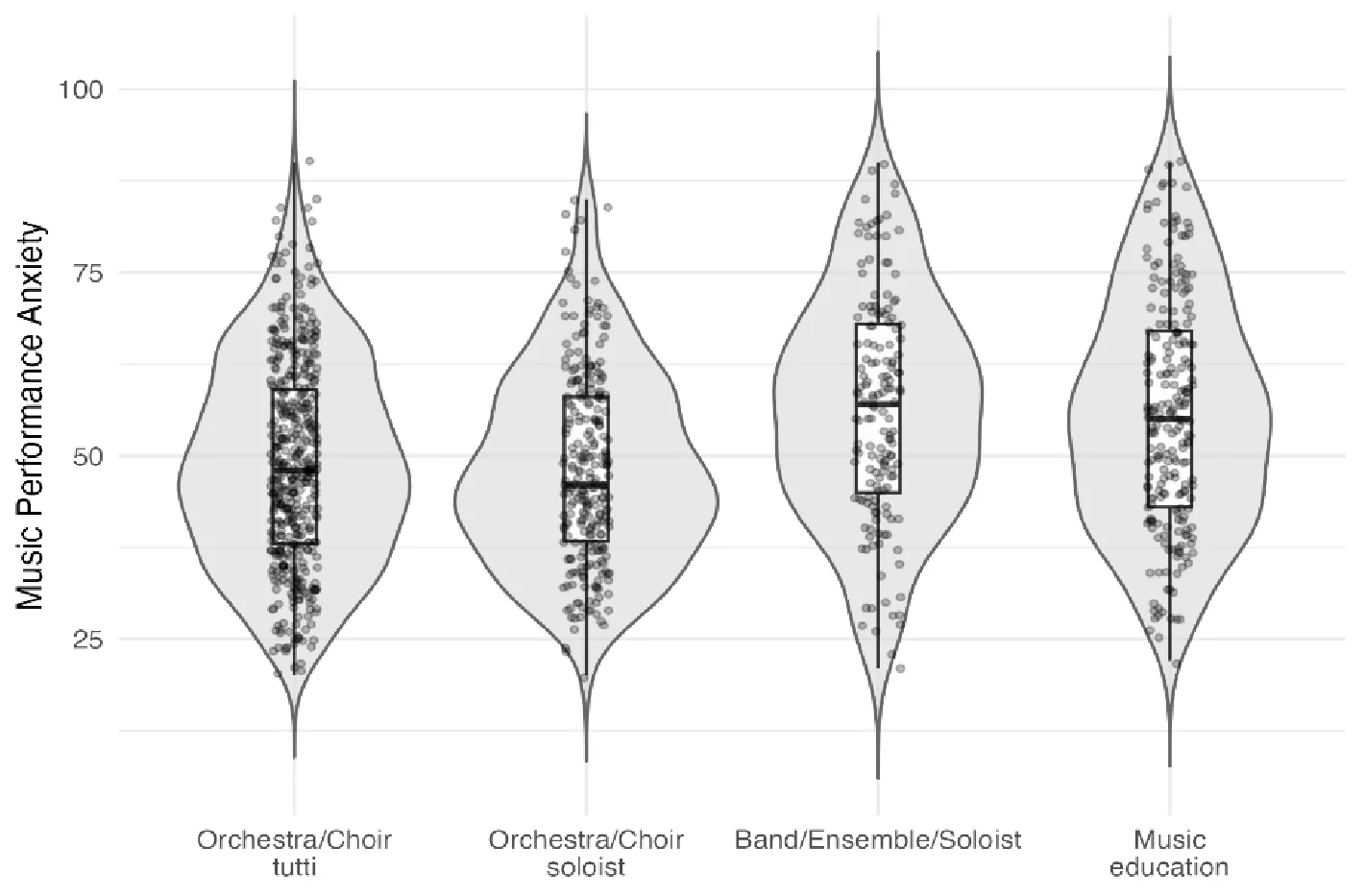
Distribution of music performance anxiety by job role at T1. Boxes show the median and interquartile range; jittered points represent individual scores. Job role categories were combined into broader groups for visual clarity; the “other” category was excluded due to its small and heterogeneous composition.

Kruskal-Wallis tests examining group differences in T1 scores across the five covariates for each of the six main constructs are summarized in Supplementary Table S1, alongside descriptive statistics for all six constructs by covariate (Supplementary Table S2). Employment status was the most consistent and strongest covariate, significant for all six constructs and yielding the largest effect observed across all tests (music performance anxiety, *η*^2^ = 0.125, moderate); gender was the second most consistent covariate, with its strongest effect also for music performance anxiety (*η*^2^ = 0.068). Job role was similarly consistent, significant for five of six constructs (largest effect for music performance anxiety, *η*^2^ = 0.055). Genre and instrument group showed the weakest and least consistent associations of the five covariates. Considered by construct rather than by covariate, resilience seemed to stand out as comparatively unrelated to demographic and occupational characteristics, reaching significance only for employment status and gender, both with small to negligible effect sizes. Figures 1–3 illustrate the distribution of T1 scores for the covariate–construct combinations with the largest effects; the remaining significant combinations are shown in Supplementary Figures S1–S12.

Dunn’s post-hoc test was used to identify which specific pairs of groups differed; full results are reported in Supplementary Table S3. For gender, women scored higher than men on both depressive symptoms (*p* < 0.001) and generalized anxiety symptoms (*p* < 0.001). For employment status, students consistently showed the least favorable profile: higher depressive symptoms and generalized anxiety symptoms than employed musicians (both *p* ≤ 0.005), higher music performance anxiety than both employed and freelance musicians (both *p* < 0.001), and lower self-esteem than both employed and freelance musicians (both *p* ≤ 0.007). For job role, the pattern was more mixed: choir soloists showed higher depressive symptoms than instrumental/voice soloists (*p* = 0.016) but lower depressive and generalized anxiety symptoms than music teachers (both *p* = 0.004) and lower depressive symptoms than orchestra soloists (*p* = 0.043), alongside higher self-esteem than orchestra soloists (*p* = 0.033).

### 3.5 Cross-sectional regressions: personal resources as predictors

Linear regression models examining resilience, self-efficacy, and self-esteem as individual predictors of T1 symptom burden, controlling for gender, genre and employment status, are presented in Table 4. All nine resource–symptom associations were significant (all *p* < 0.001) and in the expected direction, with self-esteem showing the largest unstandardized association with music performance anxiety (*b* = −6.11). Models explained between 19.5 and 34.4% of variance in symptom burden (*R*^2^). A combined model entering all three resources simultaneously was used to control for shared variance (Supplementary Table S4).

**Table 4:** Adjusted cross-sectional associations of psychological resources with mental health indicators at T1.

		Regression statistics				
	*b*					
*t*	SE	*R* ^2^	95% CI	*p*
Depressive symptoms						
Resilience	−0.2712	−15.79	0.0172	0.271	[−0.3049, −0.2375]	<0.001
Self-efficacy	−0.4127	−12.37	0.0334	0.195	[−0.4782, −0.3473]	<0.001
Self-esteem	−2.3251	−14.86	0.1564	0.246	[−2.6320, −2.0182]	<0.001
Generalized anxiety symptoms						
Resilience	−0.1985	−12.99	0.0153	0.234	[−0.2285, −0.1685]	<0.001
Self-efficacy	−0.3317	−10.81	0.0307	0.203	[−0.3919, −0.2715]	<0.001
Self-esteem	−1.7514	−12.26	0.1429	0.226	[−2.0317, −1.4711]	<0.001
Music performance anxiety						
Resilience	−0.3918	−8.57	0.0457	0.236	[−0.4815, −0.3021]	<0.001
Self-efficacy	−1.1170	−13.71	0.0815	0.299	[−1.2769, −0.9571]	<0.001
Self-esteem	−6.1139	−16.57	0.3689	0.344	[−6.8379, −5.3900]	<0.001

All models additionally control for gender, employment status, and genre (coefficients not shown). *b*, *t*, SE, CI, and *p* are based on HC3-robust standard errors. Sample size and residual d*f* (values differ slightly across the three predictor models even for the same outcome, due to differing missing-data patterns): for the resilience models, depressive symptoms: *n* = 1,060; d*f* = 1,054; generalized anxiety symptoms: *n* = 1,058; d*f* = 1,052; music performance anxiety: *n* = 1,050; d*f* = 1,044; for the self-efficacy models, depressive symptoms: *n* = 1,090; d*f* = 1,084; generalized anxiety symptoms: *n* = 1,088, d*f* = 1,082; music performance anxiety: *n* = 1,076; d*f* = 1070; for the self-esteem models, depressive symptoms: *n* = 1,103; d*f* = 1,097; generalized anxiety symptoms: *n* = 1,098; d*f* = 1,092; music performance anxiety: *n* = 1,088; d*f* = 1082.

### 3.6 Longitudinal regressions: T2 outcomes predicted by T1 baseline scores and covariates

Three linear regression models predicting each T2 symptom burden outcome from its own centered T1 baseline, plus gender, employment status, and genre, and a fourth model additionally predicting T2 psychological health from T1 physical health and T1 psychological health, are presented in Table 5. Across all four models, the T1 baseline was the dominant predictor of T2 scores (all *p* < 0.001), with models explaining between 33.8 and 67.2% of variance in T2 outcomes, indicating substantial stability of symptom levels and health status over the 12-month interval.

**Table 5:** Baseline-adjusted longitudinal regression models of mental health outcomes at T2 with covariates.

Term	*b*	*t*	SE	95% CI	*p*
Depressive symptoms (T1 → T2)					
Baseline (T1)	0.6149	10.41	0.0591	[0.4987, 0.7312]	<0.001
Gender (female)	0.4780	1.09	0.4377	[−0.3836, 1.3396]	0.276
Employment status (freelance)	0.6393	0.90	0.7084	[−0.7550, 2.0337]	0.368
Employment status (student)	1.5829	2.61	0.6074	[0.3874, 2.7785]	0.010
Genre (popular)	1.0300	1.51	0.6824	[−0.3132, 2.3732]	0.132
Generalized anxiety symptoms (T1 → T2)					
Baseline (T1)	0.6856	13.79	0.0497	[0.5877, 0.7834]	<0.001
Gender (female)	0.4595	1.10	0.4175	[−0.3623, 1.2813]	0.272
Employment status (freelance)	0.8758	1.21	0.7243	[−0.5500, 2.3015]	0.228
Employment status (student)	0.7366	1.34	0.5486	[−0.3432, 1.8165]	0.180
Genre (popular)	0.5974	1.01	0.5894	[−0.5628, 1.7577]	0.312
Music performance anxiety (T1 → T2)					
Baseline (T1)	0.8286	17.82	0.0465	[0.7371, 0.9202]	<0.001
Gender (female)	1.1256	0.94	1.2005	[−1.2381, 3.4892]	0.349
Employment status (freelance)	1.7059	1.10	1.5438	[−1.3338, 4.7455]	0.270
Employment status (student)	0.9796	0.66	1.4823	[−1.9389, 3.8981]	0.509
Genre (popular)	0.5037	0.34	1.4948	[−2.4396, 3.4469]	0.736
Physical → Psychological health					
Baseline (T1)	0.1574	2.55	0.0618	[0.0358, 0.2790]	0.011
Psychological health (T1)	0.4956	7.97	0.0622	[0.3732, 0.6180]	<0.001
Gender (female)	−0.1434	−0.72	0.1984	[−0.5339, 0.2471]	0.470
Employment status (freelance)	0.2290	0.71	0.3246	[−0.4099, 0.8678]	0.481
Employment status (student)	−0.2761	−1.14	0.2414	[−0.7512, 0.1991]	0.254
Genre (popular)	−0.3006	−1.02	0.2944	[−0.8800, 0.2789]	0.308

*b*, *t*, SE, CI, and *p* are HC3-robust. Baseline (T1) is the outcome’s own centered T1 value. The last model additionally includes T1 psychological health as a predictor. *n*, d*f*, *R*^2^: depressive symptoms *n* = 294, d*f* = 288, *R*^2^ = 0.461; generalized anxiety *n* = 288, d*f* = 282, *R*^2^ = 0.474; music performance anxiety *n* = 271, d*f* = 265, *R*^2^ = 0.672; physical health → psychological health *n* = 299, d*f* = 292, *R*^2^ = 0.338.

Beyond the baseline, employment status was associated with additional change over time only for depressive symptoms: students showed significantly higher T2 depressive symptom scores than would be expected from their T1 baseline alone (*b* = 1.58, *p* = 0.010); a joint test of the two employment-status contrasts (freelance and student) was significant, *F* (2, 288) = 3.44, *p* = 0.033.

### 3.7 Mental disorders

Overall, 22.4% of the respondents reported a currently diagnosed mental disorder, and 22.8% reported having had a diagnosed mental disorder during the past 12 months at baseline. As point and 12 month prevalence estimates were nearly identical, diagnostic categories are reported for point prevalence only. The most frequently reported current diagnoses were depressive disorders (12.6%), followed by anxiety disorders (5.1%), attention-deficit/hyperactivity disorder (1.9%), post-traumatic stress disorder (1.8%), adjustment disorders (1.4%), and eating disorders (1.3%). Less frequently reported diagnoses included obsessive-compulsive disorder (0.7%), somatoform disorders, sleep disorders, and personality disorders (0.5% each), as well as bipolar disorder, autism spectrum disorder, and other disorders (0.4% each). Several other diagnoses, including alcohol dependence, were reported by only one respondent each.

No respondent reported a specific diagnosis of music performance anxiety at baseline. Within the anxiety disorder category, social phobia, under which performance-related anxiety may clinically fall, was reported by only 4 (0.3%) respondents.

Among respondents reporting a mental disorder, 63.1% indicated that the diagnosis had been made by a psychiatrist or psychotherapist, 9.2% by a general practitioner, and 11.4% by another source, while 16.4% reported self-diagnosis.

At follow-up, the point prevalence of self-reported diagnosed mental disorders was 22.6%, and thus very similar to the baseline. The distribution of diagnostic categories was also broadly comparable to baseline; one participant explicitly reported music performance anxiety at follow-up.

### 3.8 Treatment utilization

At T1, participants were asked whether they had ever received treatment for psychological complaints (Supplementary Table S5); 50.4% indicated that they had, of whom 50.7% reported psychotherapeutic treatment, 16.5% other treatment, and 12.5% pharmacological treatment. Treatment utilization at T1 differed significantly across all five covariates: gender, *χ*^2^ (2) = 40.50, *p* < 0.001 (descriptively lowest among male musicians and highest among those identifying as diverse/other); employment status, *χ*^2^ (2) = 25.72, *p* < 0.001 (descriptively highest among freelancers); job role, *χ*^2^ (7) = 24.35, *p* < 0.001; instrument group, *χ*^2^ (9) = 22.73, *p* = 0.007; and genre, *χ*^2^ (1) = 4.08, *p* = 0.043 (descriptively higher among popular musicians). The corresponding T2 item asked whether participants had received treatment within the past 12 months: 31.0% indicated that they had.

The “other” category was detailed by participants’ self-reported free-text responses and comprised a heterogeneous range of support formats, including coaching, relaxation techniques, mental coaching for performance anxiety, hypnosis, counseling services, rehabilitation, self-help, social services, and somatic experiencing. In addition, some participants reported unmet treatment needs, such as being on psychotherapy waiting lists, in some cases for several years.

## 4 Discussion

### 4.1 Summary and interpretation of key findings

The present study provides a broad and integrative account of mental health indicators, personal resources, occupational characteristics, and treatment utilization among professional musicians and music students in Germany. Taken together, the findings point to a substantial and unevenly distributed mental health burden within this population.

The proportion of participants scoring above established cut-offs for clinically relevant symptoms was substantial. At T1, 30% scored in the moderate or severe range of generalized anxiety symptoms (GAD-7) and 32% in the moderate to severe range of depressive symptoms (PHQ-9). Although direct comparisons with population estimates are limited by differences in sampling and sample composition, it is noteworthy that these rates are higher than recent RKI Panel estimates for adults in Germany, which reported anxiety symptoms in 14% and depressive symptoms in 22% of the population using the same cut-offs (Walther et al., 2025). They are also broadly consistent with previous studies reporting elevated distress and clinically relevant symptom levels among musicians, including, for example, large survey data from Norwegian musicians (*n* = 1,607; Vaag et al., 2016a), findings from professional orchestral musicians in Australia (*n* = 377; Kenny et al., 2014), Brazilian musicians (*n* = 230; Barbar et al., 2014), and an international sample of 254 musicians from 13 countries (Loveday et al., 2023).

Within the present sample, depressive and generalized anxiety symptoms also varied across subgroups, with particularly consistent differences by employment status and additional differences by gender and genre. These patterns suggest that symptom burden among musicians is not evenly distributed, but may be shaped by specific occupational and sociodemographic conditions.

Mental health indicators showed only minor changes over the one-year period. This is consistent with longitudinal research showing that depressive and anxiety symptoms often display substantial temporal stability (Nivard et al., 2015; Conway et al., 2016) and may be especially interpreted in light of the recurrent nature of many occupational conditions relevant to musicians’ mental health, including performance pressure, career uncertainty, irregular schedules, and physical strain (Willis et al., 2024; Rousseau et al., 2026; Dobson, 2011). However, the overall stability in mean levels should not obscure subgroup-specific patterns. In particular, students showed higher depressive symptoms at follow-up than would have been expected based on their baseline levels. This finding points to a potentially vulnerable phase in musicians’ careers, in which factors such as intensive training, repeated evaluation, financial uncertainty, and the transition into professional life may contribute to increasing depressive symptom burden.

The cross-sectional findings further highlight the relevance of personal psychological resources. Resilience, self-efficacy, and self-esteem were each negatively associated with depressive symptoms, generalized anxiety symptoms, and music performance anxiety, even after accounting for gender, employment status, and genre. These findings are consistent with established psychological frameworks emphasizing the role of coping resources, perceived competence, and self-related beliefs in mental health (Bandura, 1977; Sowislo and Orth, 2013; Troy et al., 2023; Schäfer et al., 2024; Singh et al., 2022). They also resonate with research on performance-based contexts, in which repeated exposure to evaluation, comparison, and uncertainty may make self-evaluative processes particularly salient (Fernholz et al., 2019; Castiglione et al., 2018). Self-esteem showed especially strong associations with symptom burden, which is plausible in a professional context in which artistic achievement, external evaluation, and professional recognition may be closely tied to musicians’, personal identity, self-concept and self-worth (Castiglione et al., 2018). At the same time, these associations should not be interpreted unidirectionally. Meta-analytic evidence suggests that low self-esteem may contribute to subsequent depression and anxiety, while depressive symptoms may also undermine self-esteem over time (Sowislo and Orth, 2013). Moreover, low self-esteem is itself a central feature of depressive symptomatology, making conceptual and empirical overlap likely (Harrison et al., 2022). Future longitudinal and intervention studies are therefore needed to clarify whether strengthening self-related resources may reduce symptom burden, and whether improvements in mental health also lead to increases in self-esteem, perceived self-efficacy, and resilience.

Employment status emerged as one of the most consistent correlates of mental health indicators and personal psychological resources. Differences by employment status were observed across all six main constructs, with particularly pronounced associations for depressive symptoms, generalized anxiety symptoms, and music performance anxiety. This finding is consistent with the view that musicians’ mental health is closely linked to the structural conditions of their work (Willis et al., 2024; Rousseau et al., 2026; Cardoso et al., 2025). Employment status may serve as a proxy for differences in multiple domains of occupational conditions and may also covary with sociodemographic characteristics such as age (see footnote 1). Accordingly, employment status is probably best interpreted not as inherently favorable or unfavorable, but as a broad marker of differing constellations of occupational demands and resources, while recognizing substantial individual variation within each employment group. For example, freelance work may typically involve fluctuating income and career instability, which can impede longer-term financial and professional planning and create persistent uncertainty about future work (Willis et al., 2024; Willis et al., 2019; Shoebridge and Osborne, 2025). Irregular scheduling, short-notice engagements, and changing performance demands may further interfere with recovery, sleep, private life, and stable routines. Freelance musicians may also carry substantial administrative responsibilities, including contract negotiation, self-promotion, accounting, and the coordination of multiple concurrent roles, thereby adding demands that extend beyond artistic work itself. Limited access to employment-related benefits and social protections, such as paid sick leave, pension contributions, or employer-supported health services, may increase the consequences of illness or temporary reductions in work capacity. In addition, frequently changing projects, workplaces, and professional networks may reduce continuity in collegial relationships and contribute to periods of social isolation. In contrast, institutionally employed musicians may benefit from more predictable income, formal support structures, employment-related benefits, and regular contact with colleagues, all of which may reduce uncertainty and provide access to social and organizational resources. At the same time, institutional employment may involve other specific demands, including fixed performance obligations, organizational hierarchies, and limited control over schedules or artistic decisions (Willis et al., 2024; Perrier et al., 2024). Music students, in turn, may experience a distinct combination of intensive training, repeated evaluation, limited financial resources, and uncertainty regarding the transition into professional employment. The need to invest substantial time and effort without a predictable career outcome may be particularly relevant during this career stage. Taken together, these considerations may help explain why mental health indicators differed across employment-status groups in the present study. Further, the proposed mechanisms are consistent with the Job Demands-Resources model (Bakker and Demerouti, 2017; Xanthopoulou et al., 2007), the Conservation of Resources theory (Hobfoll et al., 2018), and a social-ecological perspective (McLeroy et al., 1988; Slimmen et al., 2024), which converge in emphasizing that mental health may depend on the particular configuration and interplay of occupational demands, available resources, and structural conditions across different work arrangements. This interpretation is further supported by findings among popular musicians identifying, for example, work insecurity, touring, performance, and professional relationships as distinct dimensions of occupational stress, with work insecurity showing particularly strong associations with depressive and anxiety symptoms (King et al., 2024).

Of note, differences by employment status were observed not only at baseline but also in longitudinal symptom patterns, with music students showing a relative increase in symptom burden from baseline to follow-up. This underscores the need to systematically consider employment-related aspects of musicians’ mental health in prevention and intervention and, in particular, to address mental health early in musical training through preventive and low-threshold support structures for music students.

Classical and popular musicians differed in depressive and generalized anxiety symptoms, but not in music performance anxiety or the personal-resource measures. Music performance anxiety is often discussed in relation to classical performance, conservatory training, and orchestral contexts (Fernholz et al., 2019). The absence of significant genre differences in music performance anxiety suggests that performance-related anxiety may not be confined to classical musicians, but may represent a broader vulnerability across musical fields. In contrast, differences in depressive and generalized anxiety symptoms may reflect genre-specific working conditions, career structures, income patterns, institutional support, or cultural norms around distress and help-seeking (Visser et al., 2022; Loveday et al., 2023). However, because classical musicians were overrepresented, these genre-related findings require replication in more balanced samples.

Gender was also associated with several mental health indicators and psychological resources at baseline. This is broadly consistent with population-based research showing gender differences in mental health (Salk et al., 2017; McLean et al., 2011; Kayrouz et al., 2025). In the context of professional music, however, such differences should also be considered in relation to gendered occupational structures (Kayrouz et al., 2025; Ferrari et al., 2022; Christiansen et al., 2022). Previous research and sector reports have documented persistent gender inequalities in music, particularly regarding earnings, representation, career opportunities, discrimination, and sexual harassment, while also pointing to broader structural barriers that may affect visibility, professional support, safety, and career progression within musical institutions and labor markets (Musicians’ Union, 2024; Borgström Källén and Ferm Almqvist, 2025; Smith et al., 2024). While the present study cannot disentangle these different mechanisms and aspects, its findings suggest that gender is a highly relevant dimension of occupational experience and should be considered more systematically in research, prevention, and support structures for musicians. Rather than treating gender only as a potential confounding variable, future work and support initiatives may address gender-specific experiences and needs more directly, including targeted prevention and support services.

Despite efforts to assess gender inclusively and to consider gender as a relevant dimension of musicians’ mental health, the present study was limited in their ability to account for gender diversity. Because very few participants were represented outside binary gender categories, inferential gender comparisons could only be conducted for women and men. Descriptively, however, participants with diverse gender identities showed indications of particularly high symptom burden and lower psychological resources. Although these findings cannot be interpreted as robust group differences, they are consistent with evidence that gender-diverse individuals may face distinct stressors, barriers to care, and forms of marginalization. Future studies may therefore aim to include gender-diverse musicians more adequately and to examine their mental health needs with sufficient statistical power. In addition, prevention and care structures should be sensitive to the specific needs and potential barriers faced by gender-diverse musicians, including experiences of discrimination, lack of belonging, and concerns about stigma or safety in institutional and clinical settings (Frost and Meyer, 2023).

The association between physical and psychological health represents another relevant finding: baseline physical health predicted psychological health 1 year later, even after accounting for baseline psychological health. This pattern is consistent with biopsychosocial models emphasizing the interdependence of bodily, psychological, and social factors in health and illness (Lehman et al., 2017). It also aligns with research in musicians showing that playing-related musculoskeletal problems are common and may have psychosocial consequences for performance ability, daily functioning, and well-being (Rousseau et al., 2026). In musicians, physical health problems may be particularly salient because they can directly affect performance capacity, increase occupational uncertainty, threaten professional identity, and reduce perceived control. Conversely, psychological strain may contribute to physical complaints through stress-related physiological processes, reduced recovery, sleep disruption, or maladaptive coping. The present findings cannot establish causal directionality, but they support an integrated understanding of musicians’ health in which physical and mental health are addressed together. In practical terms, musicians presenting with physical health problems may also benefit from access to psychological support, particularly when pain, fatigue, or concerns about performance capacity are accompanied by distress or functional impairment.

Only one respondent reported a current diagnosis of music performance anxiety, and only few participants reported a current diagnosis of social anxiety disorder (under which music performance anxiety is often subsumed). This appears striking given the prominent role of music performance anxiety in musicians’ mental health (Fernholz et al., 2019). It may reflect the distinction between performance anxiety as a symptom dimension and music performance anxiety as a formally recognized or self-reported diagnosis. Many musicians may experience performance-related anxiety as an occupational strain without receiving, seeking, or reporting a clinical diagnosis. Consistent with this interpretation, the distribution of the dimensional questionnaire scores indicated that a substantial proportion of participants reported pronounced performance anxiety symptoms, despite not reporting a corresponding diagnosis. Thus, the low diagnostic prevalence observed here likely underestimates the broader relevance of performance-related anxiety in this population. This underscores the importance of assessing music performance anxiety not only through diagnostic self-report, but also through symptom-based measures in prevention, counseling, and clinical care.

The point prevalence of self-reported diagnosed mental disorders in the musician sample was 22.4%, and the 12-month prevalence was 22.8%. These estimates are slightly below the 12-month prevalence of any mental disorder reported for the German adult population (27.7%; Jacobi et al., 2016), which was based on standardized diagnostic interviews. Differences also emerged for specific diagnostic categories. Depressive disorders were reported by 12.6% of the musicians, a rate somewhat higher than the German population estimate for affective disorders (9.3%). In contrast, anxiety disorders were reported by only about one third as many musicians (5.1%) as in the German adult population, where they represented the most common disorder group (15.3%). A particularly pronounced discrepancy was also observed for substance use disorders, which were reported by only one participant in the present study, compared with a German population estimate of 5.7%. Other clinically relevant diagnostic categories, including somatoform, adjustment, and personality disorders, were also reported only rarely in the present sample.

These differences should be interpreted cautiously, as they may partly reflect methodological differences between studies. In particular, the present survey assessed self-reported diagnosed mental disorders, whereas representative general population studies typically rely on standardized diagnostic interviews (Jacobi et al., 2016). Self-reported diagnoses may be affected by diagnostic recognition, access to assessment or treatment, willingness to disclose mental health information, and further aspects. Concerns about anonymity, stigma, or professional consequences may have additionally contributed to underreporting. Moreover, comparisons with the general population are limited by differences in sample composition, including age, gender distribution, education, and occupational characteristics. At the same time, the observed pattern may also point to genuine differences in mental health profiles among musicians. For example, anxiety-related distress in musicians may more often be experienced or reported in performance-specific terms, such as broadly defined music performance anxiety, rather than as clinically diagnosed anxiety disorders (Kenny, 2023). Moreover, sustained participation in performance-based careers may involve selection and socialization processes: individuals who remain in professional music may differ, for example, in coping resources, tolerance of evaluative stress, or strategies for managing anxiety-related symptoms (Brown et al., 2017; Chowdhury et al., 2017). However, because the present data do not allow clinical verification of diagnoses or causal interpretation, these explanations remain tentative and should be examined in future studies using standardized diagnostic assessments and more fine-grained measures.

Treatment utilization was substantial in the musician sample. Approximately half of participants reported having ever received psychotherapeutic or psychiatric treatment, and almost one third of the longitudinal subsample reported treatment within the past 12 months. These figures suggest that mental health difficulties and help-seeking are highly relevant within this population. Treatment utilization differed by gender, employment status, job role, instrument group, and genre, indicating that need of, access to, or use of care may not be evenly distributed across musician subgroups. Higher treatment utilization in some groups may reflect higher symptom burden, greater mental health literacy, lower stigma, or better access to services (Li et al., 2025; Fernholz et al., 2025; Vaag et al., 2016b; Vaag et al., 2021). Conversely, lower utilization should not be interpreted as lower need. It may reflect barriers such as time constraints, financial limitations, lack of specialized services, stigma, or concerns that disclosure could affect professional opportunities. Future research may therefore examine not only whether musicians use treatment, but also whether the support musicians receive is perceived as adequate, which barriers prevent access to care, and how perceived need relates to actual service utilization.

### 4.2 Practical significance and implications for prevention and intervention

The practical and clinical importance of the findings should be considered alongside their statistical significance. Given the large baseline sample, even small associations and subgroup differences could reach statistical significance. The strongest indications of practical relevance probably arise from the substantial proportions of participants exceeding established screening thresholds for depressive and generalized anxiety symptoms, the frequent use of mental health treatment, and reports of unmet treatment needs. Together, these findings indicate a need for accessible prevention, assessment, and care within professional music and higher music education. The subgroup findings provide additional information about contexts in which support may be particularly relevant. Most effects were negligible or small; moderate differences emerged for employment status and gender in relation to music performance anxiety. Gender, genre, employment status, and other characteristics may therefore inform context-sensitive outreach and service provision, but should be considered alongside symptom burden, functional impairment, perceived need, barriers to care, and individual circumstances. The present analyses alone cannot determine to what extent the observed mean differences translate into clinically important differences in daily or professional functioning or treatment need. The associations between psychological resources and symptom burden should also be interpreted in light of the study’s methodological characteristics. Psychological resources showed consistent small-to-moderate associations with symptom burden, supporting their potential practical relevance. However, these findings do not establish that strengthening such resources would lead to clinically meaningful symptom improvement. Given the cross-sectional observational design, the direction of these associations remains unclear. Resilience, self-efficacy, and self-esteem may therefore be regarded as plausible intervention targets whose clinical relevance requires confirmation through intervention studies.

The overall pattern of findings points to the relevance of a multilevel approach to prevention and intervention. From the perspectives of the Job Demands-Resources model (Bakker and Demerouti, 2017; Xanthopoulou et al., 2007) and Conservation of Resources theory (Hobfoll et al., 2018), the resource associations suggest potential benefits of strengthening personal resources, whereas the employment-related differences highlight the importance of reducing occupational demands and protecting access to resources such as time, energy, social support, financial security, and perceived control. A social-ecological perspective further extends this approach by emphasizing complementary measures at individual, interpersonal, institutional, and sectoral levels (McLeroy et al., 1988; Slimmen et al., 2024).

Music universities and conservatories may represent particularly relevant settings. For example, low-threshold health programs could integrate mental health literacy, stress and recovery management, support for music performance anxiety, and training in adaptive coping and psychological resources. Some musician-specific health programs have shown improvements in health knowledge, attitudes, and preventive behavior, although evidence for broader mental health effects remains limited (Fernholz et al., 2019; Brehm et al., 2026). Preliminary findings also support the feasibility of, for example, mindfulness-based, performance-psychology, cognitive-behavioral, and acceptance-based interventions in music education settings (Czajkowski et al., 2022; Fernholz et al., 2019; Clarke et al., 2020). Voluntary and confidential screening for mental health burden may complement such programs, particularly during musical training and the transition into professional work, provided that appropriate follow-up assessment, referral pathways, and treatment options are available and that screening results remain strictly separate from admissions, examinations, casting, and employment decisions. Peer-support and mentoring initiatives may provide an additional low-threshold route to support by reducing isolation and facilitating help-seeking. This is consistent with evidence that social relationships and institutional support influence health and access to care in conservatoire settings (Perkins et al., 2017). Such initiatives should complement rather than replace professional assessment and treatment. Institutions could, for example, provide confidential counseling, clear referral pathways, and training for teachers, supervisors, and other staff in recognizing and responding appropriately to distress. Occupational interventions may additionally address workload, scheduling, recovery opportunities, touring, performance pressure, and physical strain. Initial studies indicate that musician-specific workplace and multimodal wellness programs are feasible and may improve selected health outcomes, although evidence for sustained mental health effects remains limited (Roos et al., 2024; Bertsch et al., 2026). Because freelance musicians may have limited access to institution-based services, support may need to be also provided through professional associations, unions, funding bodies, or specialized musicians’ health services.

### 4.3 Limitations

Several limitations should be considered when interpreting the findings. First, the study used an observational survey design, and most analyses were based on cross-sectional baseline data. Accordingly, the findings do not allow causal conclusions and may be influenced by unmeasured third variables, selection effects, or other confounding factors. Although the longitudinal component provides initial insights into change over time, it was limited to two measurement points and based on a substantially smaller subsample than the baseline assessment. This restricts conclusions about temporal dynamics and within-person change.

Second, the study used a non-probabilistic convenience sampling approach. Thus, although recruitment was broad and nationwide, the sample cannot be assumed to be representative of the overall population of professional musicians and music students. For example, participation may have been more likely among individuals with greater perceived relevance of the survey topic, stronger institutional or professional network connections, or greater availability to complete an online survey. In addition, some subgroups were small or unevenly distributed, for example with regard to musical genre, where classical musicians were overrepresented. This may have reduced statistical power and limited the robustness of sociodemographic and profession-related comparisons. Moreover, the small number of participants identifying as gender-diverse or not reporting their gender precluded their inclusion in inferential analyses. Furthermore, while the study intentionally focused on associations and subgroup differences within musicians, the absence of a non-musician comparison group limits the extent to which the findings can be interpreted in relation to other occupational groups or the general population. In addition, because the study was conducted in Germany, its findings may not fully generalize to other countries. Consistent with a social-ecological perspective (McLeroy et al., 1988; Slimmen et al., 2024), national differences in music education, cultural funding, employment and labor markets, labor protection, social security, health-care provision, broader cultural conditions, and other dimensions may shape demands and resources (Brehm et al., 2026). For example, prevalence estimates and treatment utilization rates may therefore be particularly sensitive to national context. However, occupational factors such as employment insecurity, performance-related pressures, and limited institutional support are not unique to Germany, although their prevalence and impact may vary across countries. For example, studies conducted in European countries (Brehm et al., 2026; Vaag et al., 2016a; Aalberg et al., 2019; Kegelaers et al., 2021; Kegelaers and Gibney, 2025; Musgrave et al., 2025), the United States (King et al., 2024), Australia (Shoebridge and Osborne, 2025; Kenny et al., 2014), Japan (Takagi et al., 2026), and other countries (Loveday et al., 2023) point to related occupational challenges across different national settings. Future large-scale cross-national studies designed for direct comparison are needed to distinguish broadly shared occupational patterns from those specific to national contexts.

Third, professional music is often characterized by overlapping roles and heterogeneous working realities (Cardoso et al., 2025; Willis et al., 2024). Participants may simultaneously belong to several categories, for example by combining freelance work, institutional employment, teaching, project-based performance, and activity across multiple genres. Although pragmatic single-category classifications were necessary to ensure interpretable quantitative analyses, they inevitably simplify the complexity and fluidity of musicians’ professional lives.

Fourth, given the fragmented evidence base, the study adopted a broad analytic approach, which necessarily involved multiple subgroup and association analyses and increased the risk of false-positive findings.

Fifth, participant dropout during the two assessments, substantial longitudinal attrition from 1,392 participants at baseline to 327 participants at follow-up, and item-level nonresponse resulted in progressively smaller and varying numbers of valid cases across variables and analyses. This may have reduced statistical power, especially in subgroup and longitudinal analyses and for the detection of smaller effects. Retention also differed by genre, with participants from popular music genres being less likely to complete the follow-up assessment. Popular musicians may therefore be underrepresented in the longitudinal sample, which should be considered particularly when interpreting genre-related findings and the overall longitudinal estimates. Missingness may additionally have been related to unmeasured characteristics or to changes in mental health or occupational circumstances occurring after baseline. For example, participants experiencing increasing mental health burden, occupational instability, or time constraints may have been less likely to complete the follow-up, potentially leading to an underestimation of adverse longitudinal changes. Conversely, participants for whom mental health burden was particularly salient may have been more likely to remain in the study, which could have influenced estimates in the opposite direction. The longitudinal findings, including the observed stability of most mental health indicators and psychological resources, should therefore be interpreted cautiously as estimates based on the retained subsample rather than as definitive evidence of stability in the original sample.

Sixth, most constructs were assessed using brief measures in order to maintain feasibility and reduce participant burden. For example, global self-esteem and current mental and physical health were assessed with single-item measures, which limited psychometric precision and precluded a more differentiated assessment of these constructs. For the same reason, some potentially relevant constructs and symptom domains, such as obsessive-compulsive symptoms or eating disorder symptoms, were not included, and potential mechanisms, mediating variables, and contextual factors could not be examined in detail. Moreover, although the study assessed a broad range of variables, not all of them could be incorporated into the analyses. This was due to model complexity, statistical power considerations, and the need to limit additional Type I error inflation given the already large number of statistical tests.

Seventh, all data were based on self-report, which may have introduced recall bias, social desirability bias, common-method variance, and variability in item interpretation. Self-reported mental disorders were not verified using clinical interviews or diagnostic records; therefore, prevalence estimates should be interpreted with caution. Likewise, treatment utilization was self-reported and did not capture treatment quality, duration, indication, or other details.

## 5 Conclusion

Despite its limitations, the present study provides a broad and differentiated picture of mental health indicators, psychological resources, and treatment utilization among musicians. Overall, the findings indicate that mental health in this population cannot be understood solely in terms of individual vulnerability or isolated symptom domains. Rather, depressive symptoms, anxiety, music performance anxiety, psychological resources, treatment utilization, and professional characteristics appear to form a complex pattern that is embedded in the specific demands and characteristics of musical training and professional practice. Prevention and intervention efforts may therefore benefit from combining resource-oriented approaches, such as strengthening self-esteem, self-efficacy, and resilience, with attention to occupational conditions, including performance pressure, recovery opportunities, institutional support, and career insecurity. The findings also highlight the importance of early and accessible support within music education, as well as stigma-sensitive mental health services that are informed by the realities of professional music-making.

By providing an integrative account of mental health indicators, resources, and professional characteristics in a heterogeneous musician sample, the present study offers an important step toward a more systematic and contextualized evidence base. Building on this work, further longitudinal, comparative, and cross-national research, together with randomized controlled trials of preventive and clinical interventions, may help consolidate these findings, clarify underlying mechanisms, and translate them into targeted, evidence-based strategies to support musicians’ mental health.

## Data Availability

The raw data supporting the conclusions of this article will be made available by the authors, without undue reservation.

## Footnotes

1 Brehm, A. S., Plag, J., Schmidt, A., Petzold, M. B., Bendau, A., and Fernholz, I. (under review). Buffering the pressure: mental toughness as a moderator of the link between perfectionism and depressive symptoms in professional musicians. *Musicae Sci.*

2 Separate analyses of the baseline dataset have addressed specific research questions on substance use (Bendau et al., 2026) and mental toughness (see footnote 1). The present study represents the main integrative analysis of the project, combining mental health indicators, psychological resources, subgroup differences, and longitudinal data.
