# Supplementary material for: What plays beneath: mental health burden, personal resources, and treatment utilization among professional musicians and music students
Source: Front Psychol. 2026 Aug 21;17:1924653. doi: 10.3389/fpsyg.2026.1924653 (PMC13541520; doi:10.3389/fpsyg.2026.1924653)

Supplement

What Plays Beneath: Mental Health Burden, Personal Resources, and Treatment Utilization Among Professional Musicians and Music Students

Table S1. Kruskal-Wallis tests of differences in T1 constructs (symptom severity and psychological resources) across demographic and occupational groups.

	n	H	df	η^2H	p
Depressive Symptoms					
Gender	1301	19.89	2	0.014	<.001
Genre	1306	23.31	1	0.017	<.001
Employment Status	1287	97.97	2	0.075	<.001
Job Role	1305	54.15	7	0.036	<.001
Instrument Group	1306	16.17	9	0.006	0.063
Generalized Anxiety Symptoms					
Gender	1293	31.73	2	0.023	<.001
Genre	1297	28.28	1	0.021	<.001
Employment Status	1278	116.24	2	0.090	<.001
Job Role	1297	47.71	7	0.032	<.001
Instrument Group	1297	19.81	9	0.008	0.019
Music Performance Anxiety					
Gender	1153	79.78	2	0.068	<.001
Genre	1158	2.33	1	0.001	0.127
Employment Status	1147	145.11	2	0.125	<.001
Job Role	1157	70.49	7	0.055	<.001
Instrument Group	1158	30.66	9	0.019	<.001
Resilience					
Gender	1100	6.15	2	0.004	0.046
Genre	1105	0.38	1	-0.001	0.539
Employment Status	1094	16.87	2	0.014	<.001
Job Role	1104	11.95	7	0.005	0.102
Instrument Group	1105	8.26	9	-0.001	0.508
Self-Efficacy					
Gender	1131	15.19	2	0.012	<.001
Genre	1136	0.58	1	0.000	0.448
Employment Status	1125	8.11	2	0.005	0.017
Job Role	1136	23.18	7	0.014	0.002
Instrument Group	1136	11.70	9	0.002	0.231
Self-Esteem					
Gender	1145	31.43	2	0.026	<.001
Genre	1150	0.02	1	-0.001	0.897
Employment Status	1138	18.59	2	0.015	<.001
Job Role	1149	33.11	7	0.023	<.001
Instrument Group	1150	15.32	9	0.006	0.083

Note. η^2H denotes eta-squared based on the Kruskal-Wallis H statistic and was calculated as $\eta^2H = (H - k + 1)/(n - k)$, where k is the number of groups (Tomczak & Tomczak, 2014). Values of $\eta^2H \geq .01$, $\geq .06$, and $\geq .14$ were interpreted as approximate benchmarks for small, moderate, and large effects, respectively, following thresholds for ANOVA-based eta-squared.

Table S2. Descriptive statistics for T1 variables (symptom severity and psychological resources) by demographic and occupational characteristics.

		Depressive Symptoms		General. Anxiety Symptoms		Music Perform. Anxiety		Resilience		Self-Efficacy		Self-Esteem	
	n	M	SD	M	SD	M	SD	M	SD	M	SD	M	SD
Gender													
Male	547	7.26	5.26	6.72	4.76	46.53	13.35	59.52	9.25	28.95	4.82	3.35	1.01
Female	810	8.31	5.49	8.00	4.89	54.10	14.68	60.63	8.87	27.98	4.69	3.04	0.99
Diverse/Other	22	11.38	6.18	10.50	5.30	61.80	15.76	57.95	5.82	26.95	4.25	2.75	1.07
Genre													
Classical	985	7.53	5.36	7.10	4.79	50.75	14.50	60.30	8.89	28.25	4.76	3.16	1.00
Popular	407	8.97	5.53	8.62	4.98	52.44	15.19	59.85	9.28	28.61	4.75	3.14	1.04
Employment Status													
Employed	724	6.64	5.00	6.23	4.46	46.90	13.38	61.19	8.58	28.67	4.58	3.26	0.97
Freelance	263	9.16	5.86	8.62	5.07	52.65	15.32	59.46	9.32	28.07	5.25	3.13	0.98
Student	379	9.61	5.42	9.35	4.89	59.24	13.36	58.51	9.39	27.85	4.67	2.95	1.09
Job Role													
Orchestra tutti	412	7.23	5.08	6.88	4.54	50.54	13.86	60.35	8.56	27.94	4.42	3.12	0.97
Choir tutti	129	7.87	5.00	6.64	4.56	44.24	13.60	59.95	8.93	28.53	4.89	3.39	0.95
Orchestra solo	241	6.92	5.45	6.85	5.07	48.54	12.94	61.54	8.63	29.16	4.70	3.26	1.00
Choir solo	57	8.70	5.67	8.29	4.87	46.98	13.64	61.35	8.68	29.89	3.66	3.62	0.89
Instrum./voice solo	122	10.25	6.11	9.25	5.19	57.60	16.42	58.54	9.28	27.43	5.05	2.81	1.03
Ensemble	78	10.28	5.12	9.64	4.83	54.02	13.24	58.04	9.95	28.08	4.71	3.07	1.06
Music teaching	265	8.09	5.47	7.87	4.89	55.55	15.72	59.74	9.56	28.12	5.16	3.07	1.04
Other	79	8.07	5.47	8.21	4.97	50.95	13.60	60.18	9.08	28.90	5.07	3.13	1.00
Instrument Group													
Strings (bowed)	392	7.61	5.29	7.26	4.80	51.67	14.39	60.28	8.67	27.91	4.48	3.04	1.00
Winds (woodwinds)	201	7.73	5.56	7.43	4.76	50.66	13.89	60.47	8.37	28.01	4.45	3.07	0.97
Brass	127	6.94	5.13	6.27	4.50	50.45	12.31	60.48	8.69	28.90	4.88	3.25	0.92
Percussion	57	8.04	6.30	7.94	5.32	45.83	13.97	57.31	12.64	28.50	5.36	3.41	1.12
Singing	222	8.47	5.41	7.83	4.88	48.59	15.02	60.34	8.21	29.04	4.39	3.30	1.00
Keyboard	142	8.15	5.76	8.04	5.32	56.94	16.47	59.56	10.07	28.12	5.38	3.09	1.07
Plucked strings	42	7.67	5.51	7.08	5.17	51.81	15.40	58.67	8.71	28.37	5.01	3.03	0.96
Multiple instruments	146	8.59	5.15	8.35	4.72	52.11	14.75	61.54	9.27	28.61	5.25	3.20	1.06
Other	17	7.94	4.07	7.33	3.06	52.08	11.83	58.67	7.48	27.69	2.93	3.23	0.60
No response	46	9.90	6.48	8.47	5.92	47.79	15.34	59.15	9.00	28.48	5.56	3.33	1.00

Note. n refers to the number of valid responses in each demographic or occupational category.

Table S3. Significant Bonferroni-adjusted Dunn pairwise comparisons at T1.

Covariate	Comparison (higher group > lower group)[1]	|Z|	p (adjusted)
Depressive Symptoms			
Gender	Female > Male	3.94	<.001
Employment Status	Student > Employed	3.14	0.003
Job Role	Instrumental/voice solo > Choir solo	3.25	0.016
Job Role	Choir solo > Music teaching	3.61	0.004
Job Role	Choir solo > Orchestra solo	2.96	0.043
Generalized Anxiety Symptoms			
Gender	Female > Male	4.62	<.001
Employment Status	Student > Employed	2.96	0.005
Job Role	Choir solo > Music teaching	3.61	0.004
Music Performance Anxiety			
Employment Status	Student > Employed	4.14	<.001
Employment Status	Student > Freelance	3.77	<.001
Job Role	Other > Orchestra tutti	2.98	0.041
Instrument Group	Keyboard > Strings (bowed)	3.06	0.050
Self-Esteem			
Employment Status	Employed > Student	2.90	0.006
Employment Status	Freelance > Student	2.83	0.007
Job Role	Choir solo > Orchestra solo	3.04	0.033

Note. Dunn tests were conducted following significant Kruskal-Wallis tests at T1. Only the 15 pairwise comparisons that remained significant after Bonferroni adjustment are shown; the full set of comparisons is available on request. Comparisons are presented with the higher-ranked group first, and |Z| denotes the absolute value of the Dunn test statistic.

.

Table S4. Adjusted cross-sectional regression models of T1 symptom severity with resilience, self-efficacy, and self-esteem entered simultaneously and covariates included.

	b	t	SE	95% CI	p
Depressive Symptoms					
Resilience	-0.1842	-8.19	0.0225	[-0.2283, -0.1400]	<.001
Self-Efficacy	-0.0465	-1.09	0.0428	[-0.1304, 0.0374]	0.277
Self-Esteem	-1.3190	-6.55	0.2013	[-1.7140, -0.9239]	<.001
Gender (Female)	0.6123	2.06	0.2974	[0.0287, 1.1959]	0.040
Freelance	1.7659	4.08	0.4328	[0.9165, 2.6152]	<.001
Student	1.6702	4.78	0.3496	[0.9842, 2.3563]	<.001
Genre (Popular)	0.5544	1.53	0.3632	[-0.1583, 1.2670]	0.127
Generalized Anxiety Symptoms					
Resilience	-0.1199	-5.92	0.0202	[-0.1596, -0.0802]	<.001
Self-Efficacy	-0.0890	-2.12	0.0419	[-0.1713, -0.0067]	0.034
Self-Esteem	-0.9673	-4.85	0.1994	[-1.3585, -0.5761]	<.001
Gender (Female)	1.0495	3.82	0.2745	[0.5109, 1.5882]	<.001
Freelance	1.6633	4.14	0.4022	[0.8741, 2.4525]	<.001
Student	2.0417	5.93	0.3442	[1.3664, 2.7171]	<.001
Genre (Popular)	0.9073	2.63	0.3445	[0.2313, 1.5832]	0.009
Music Performance Anxiety					
Resilience	0.0179	0.34	0.0523	[-0.0848, 0.1206]	0.733
Self-Efficacy	-0.5825	-5.63	0.1034	[-0.7855, -0.3795]	<.001
Self-Esteem	-4.5107	-9.37	0.4812	[-5.4548, -3.5665]	<.001
Gender (Female)	4.9858	6.62	0.7529	[3.5084, 6.4633]	<.001
Freelance	5.2482	4.59	1.1425	[3.0063, 7.4901]	<.001
Student	10.1647	11.15	0.9116	[8.3759, 11.9536]	<.001
Genre (Popular)	-0.5054	-0.53	0.9613	[-2.3918, 1.3810]	0.599

Note. HC3 heteroskedasticity-robust SEs are reported; t statistics, 95% CIs, and p values are based on these SEs. Reference categories were male for gender, employed for employment status, and classical for genre. Participants identifying as diverse/other were excluded from the regression analyses because the group size was insufficient for reliable estimation. Model statistics were as follows: depressive symptoms, n = 1045, residual df = 1037, R^2 = .319; generalized anxiety symptoms, n = 1045, residual df = 1037, R^2 = .275; music performance anxiety, n = 1035, residual df = 1027, R^2 = .360.

Table S5. Treatment utilization at T1 and T2 by demographic and occupational characteristics.

	T1 (lifetime)						T2 (past 12 months)		
	n	n (Yes)	% (Yes)	% Psycho-therap.	% Pharma-cological	% Other	n	n (Yes)	% (Yes)
Gender									
Male	541	220	40.7	50.0	15.9	14.1	114	29	25.4
Female	799	452	56.6	51.1	10.8	17.5	191	63	33.0
Diverse/Other	21	17	81.0	47.1	11.8	23.5	9	5	55.6
Genre									
Classical	973	473	48.6	48.6	11.8	16.3	243	68	28.0
Popular	394	216	54.8	55.1	13.9	17.1	73	30	41.1
Employment Status									
Employed	717	351	49.0	46.2	13.4	18.8	167	43	25.7
Freelance	258	164	63.6	48.8	14.0	17.1	68	27	39.7
Student	373	162	43.4	63.0	7.4	11.1	77	26	33.8
Job Role									
Orchestra tutti	405	204	50.4	42.2	11.8	19.6	100	26	26.0
Choir tutti	129	68	52.7	45.6	14.7	13.2	33	10	30.3
Orchestra solo	240	91	37.9	52.7	9.9	12.1	55	12	21.8
Choir solo	57	25	43.9	60.0	32.0	24.0	17	5	29.4
Instrument./voice solo	118	63	53.4	57.1	15.9	15.9	25	15	60.0
Ensemble	77	44	57.1	70.5	13.6	11.4	14	7	50.0
Music teaching	263	148	56.3	47.3	9.5	20.3	58	17	29.3
Other	77	46	59.7	69.6	10.9	6.5	14	6	42.9
Instrument Group									
Strings (bowed)	385	184	47.8	46.7	12.0	14.1	86	23	26.7
Winds (woodwinds)	200	94	47.0	51.1	5.3	20.2	50	15	30.0
Brass	126	52	41.3	48.1	15.4	15.4	30	7	23.3
Percussion	57	24	42.1	45.8	8.3	20.8	9	1	11.1
Singing	220	129	58.6	50.4	14.7	17.1	58	22	37.9
Keyboard	141	73	51.8	64.4	13.7	15.1	37	11	29.7
Plucked strings	42	25	59.5	60.0	20.0	8.0	8	3	37.5
Multiple instruments	144	86	59.7	41.9	12.8	20.9	33	14	42.4
Other	17	9	52.9	66.7	0.0	11.1	2	1	50.0
No response	35	13	37.1	76.9	30.8	15.4	3	1	33.3

Note. T1 treatment utilization refers to lifetime use, whereas T2 treatment utilization refers to use during the past 12 months. Percentages reporting any treatment are based on the number of valid respondents within each category. T1 treatment-type percentages for psychotherapy, pharmacological treatment, and other treatment are based on participants reporting lifetime treatment. Because treatment types were assessed separately and multiple responses were possible, these percentages do not sum to 100%.

Supplementary Figures S1-S12.

Distributions of T1 study variables across demographic and occupational groups.

Note (applies to all Figures S1–S12). Boxes represent the median and interquartile range, and jittered points individual observations. The figures display statistically significant differences across demographic and occupational groups that are not presented in the main article. In figures examining job role, categories were combined into broader groups to improve visual clarity; the "other" category was excluded because of its small and heterogeneous composition.

Figure S1. Distribution of depressive symptoms by job role at T1.

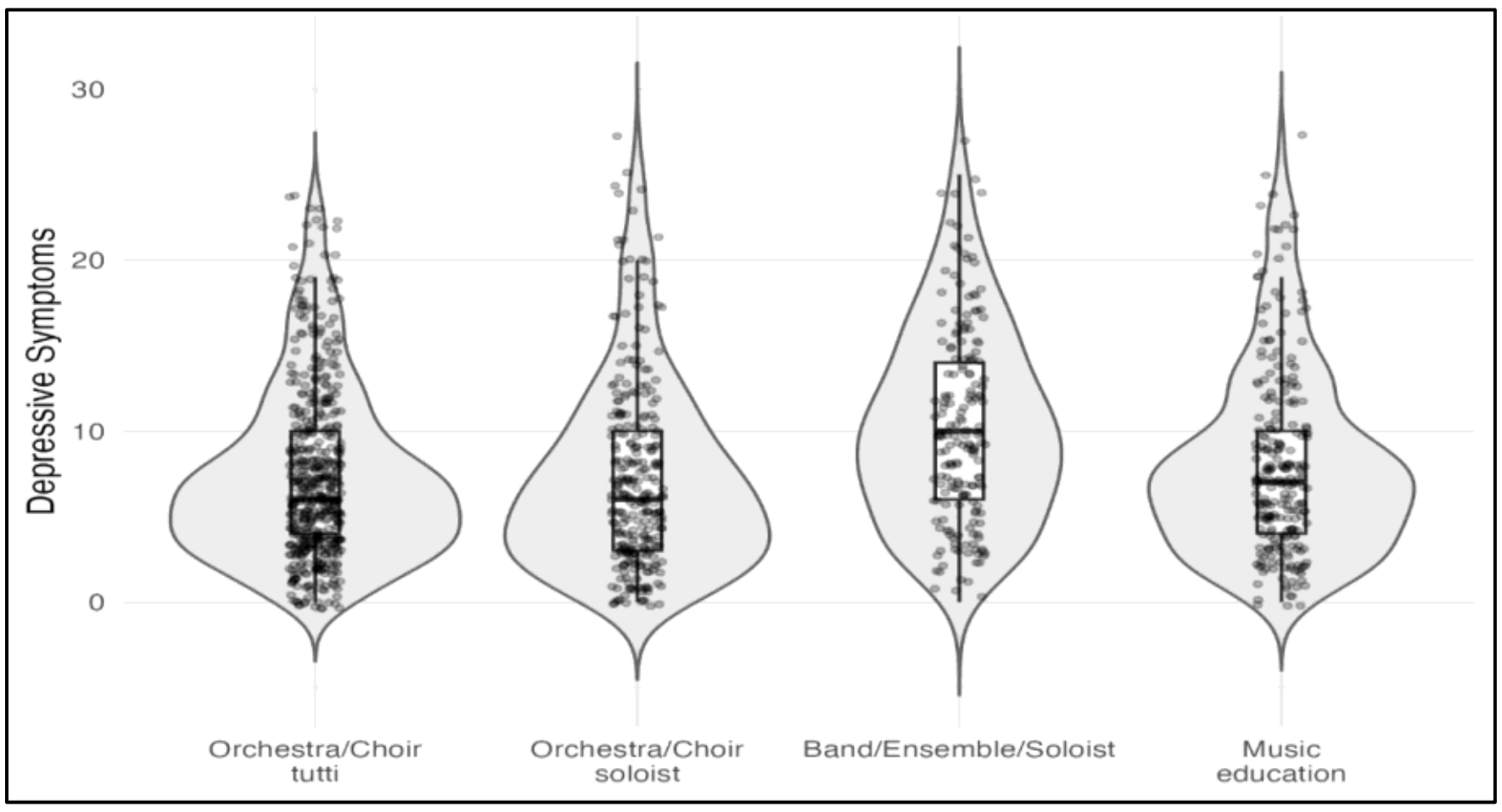

Figure S2. Distribution of generalized anxiety symptoms by job role at T1.

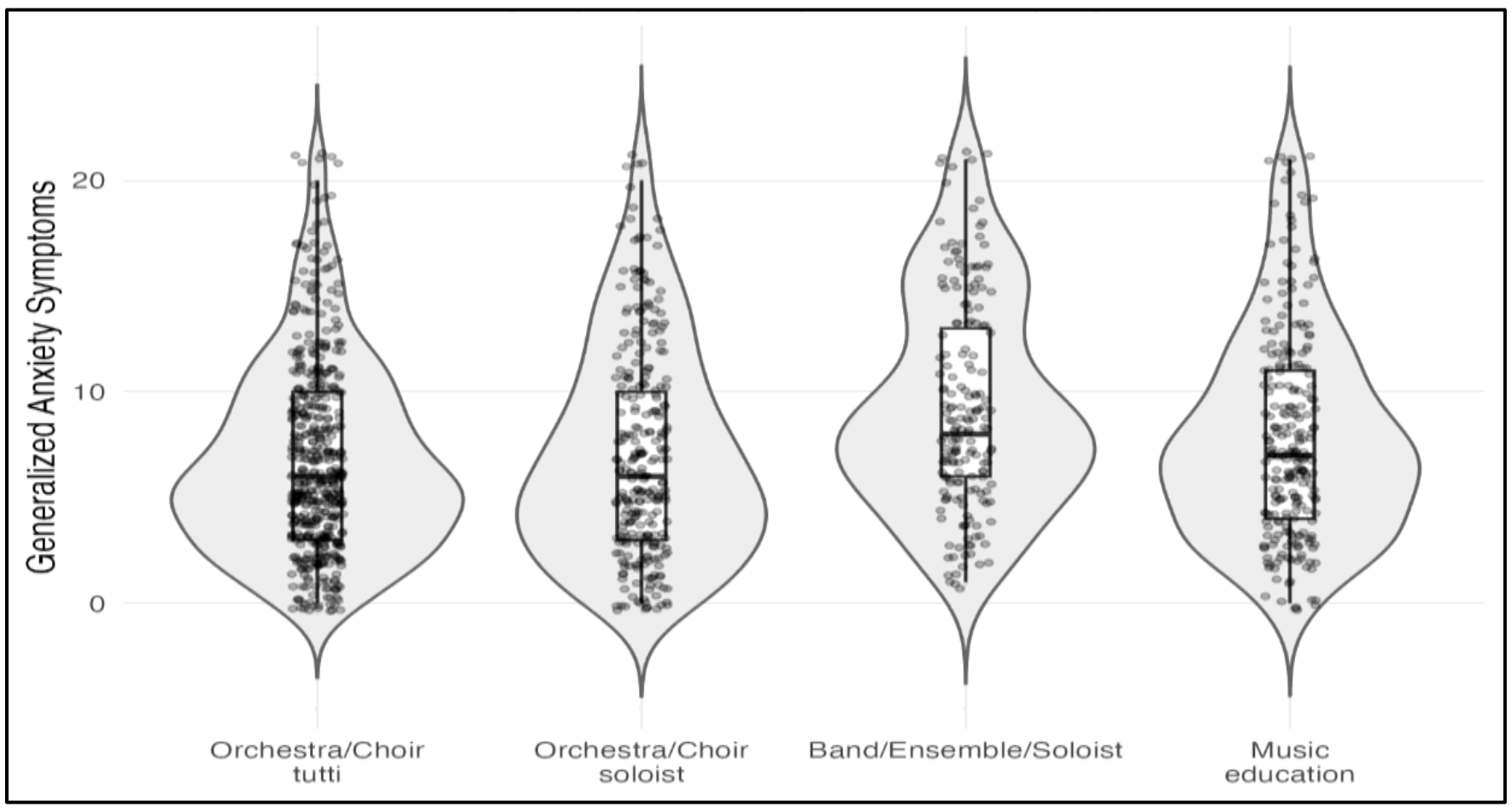

Figure S3. Distribution of generalized anxiety symptoms by gender at T1.

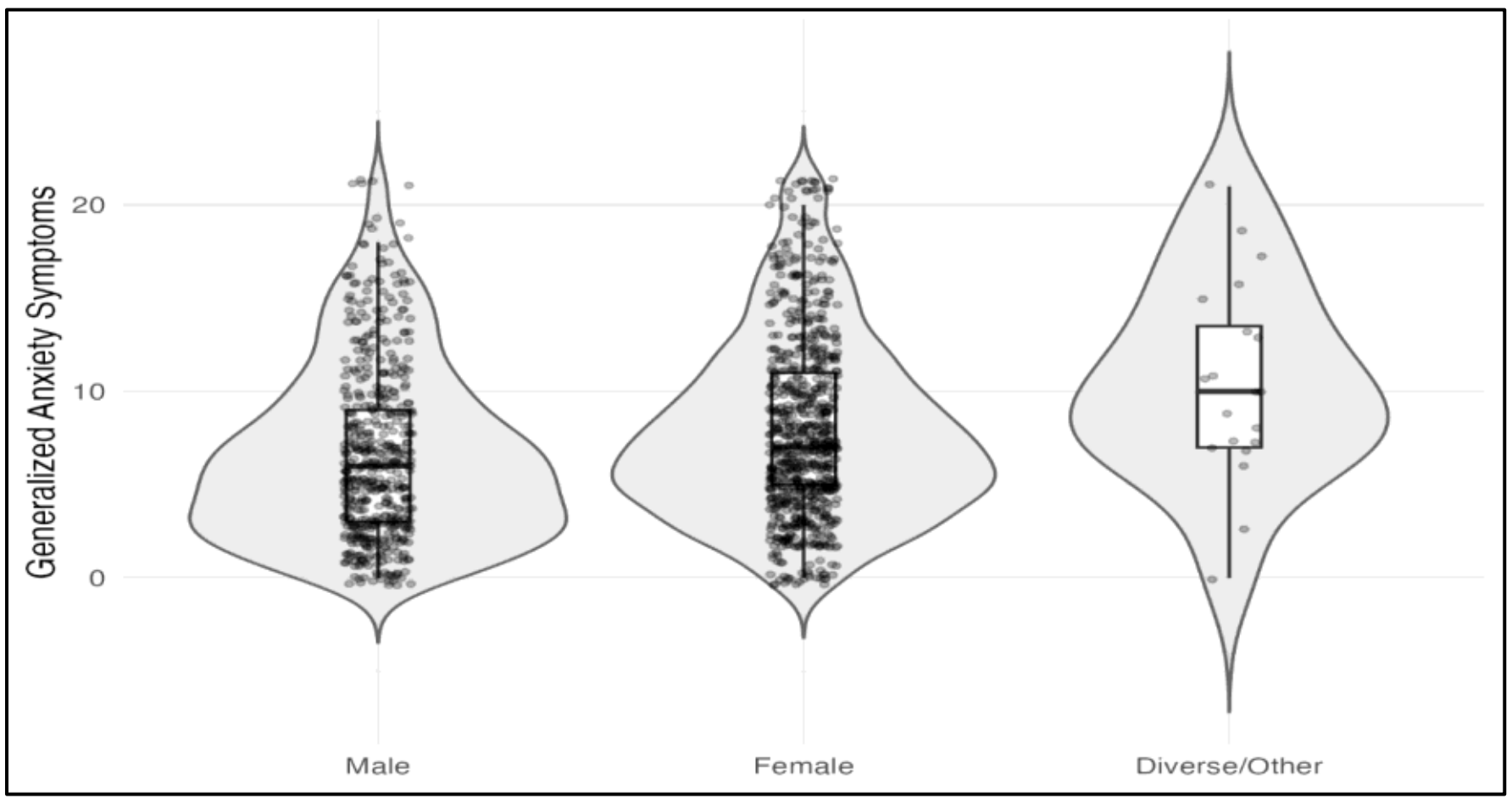

Figure S4. Distribution of self-esteem by job role at T1.

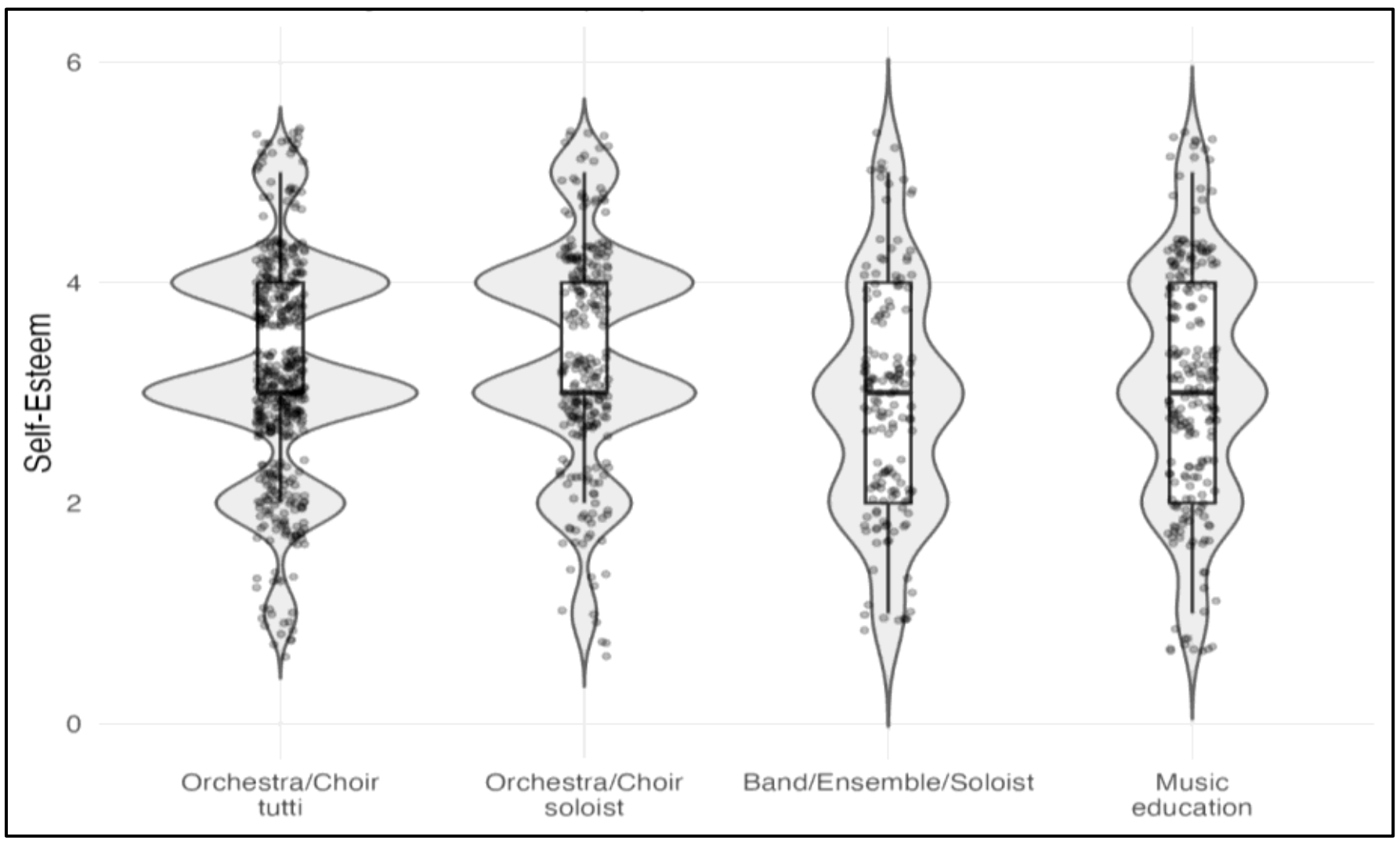

Figure S5. Distribution of generalized anxiety symptoms by genre at T1.

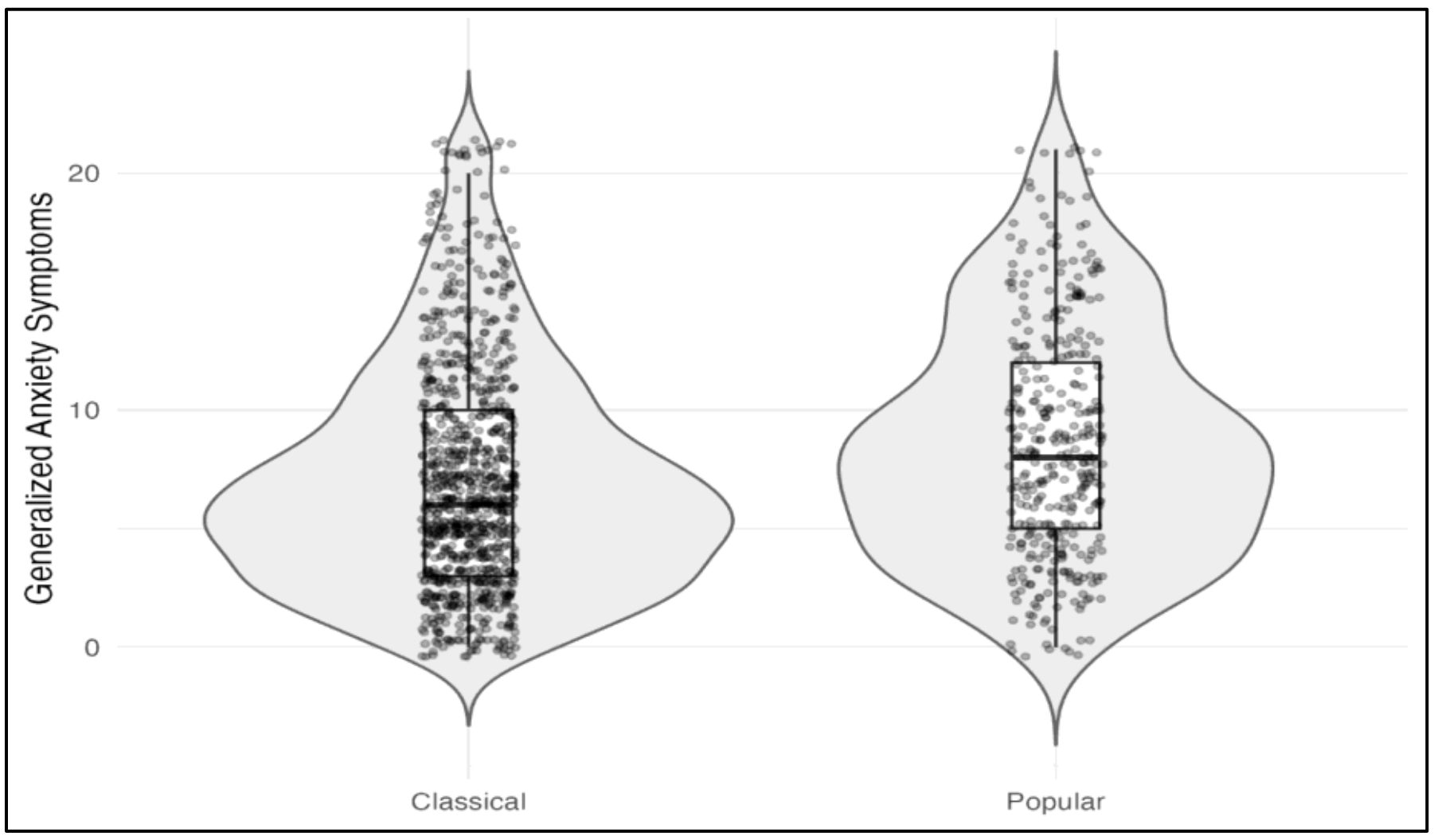

Figure S6. Distribution of depressive symptoms by genre at T1.

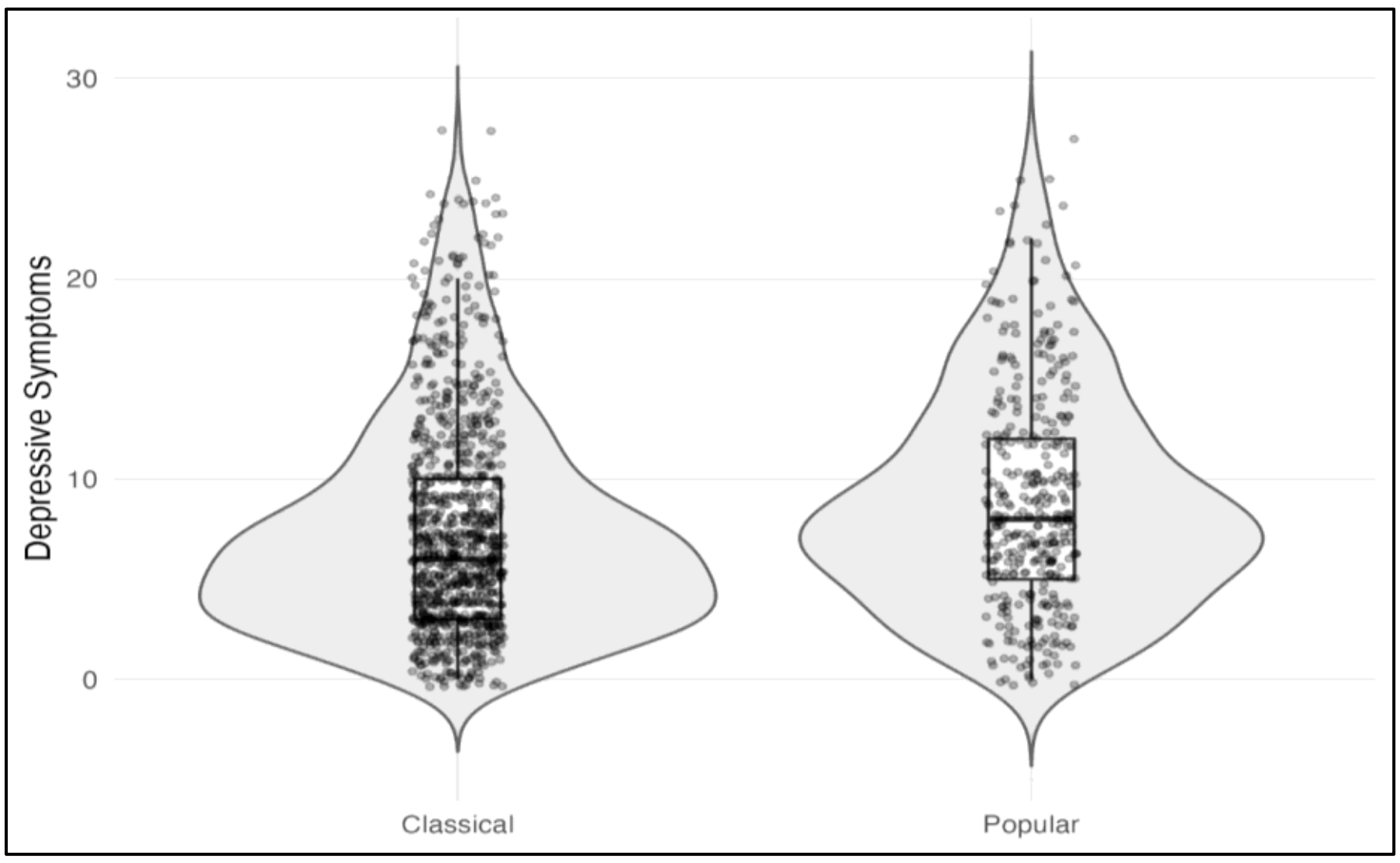

Figure S7. Distribution of self-esteem by employment status at T1.

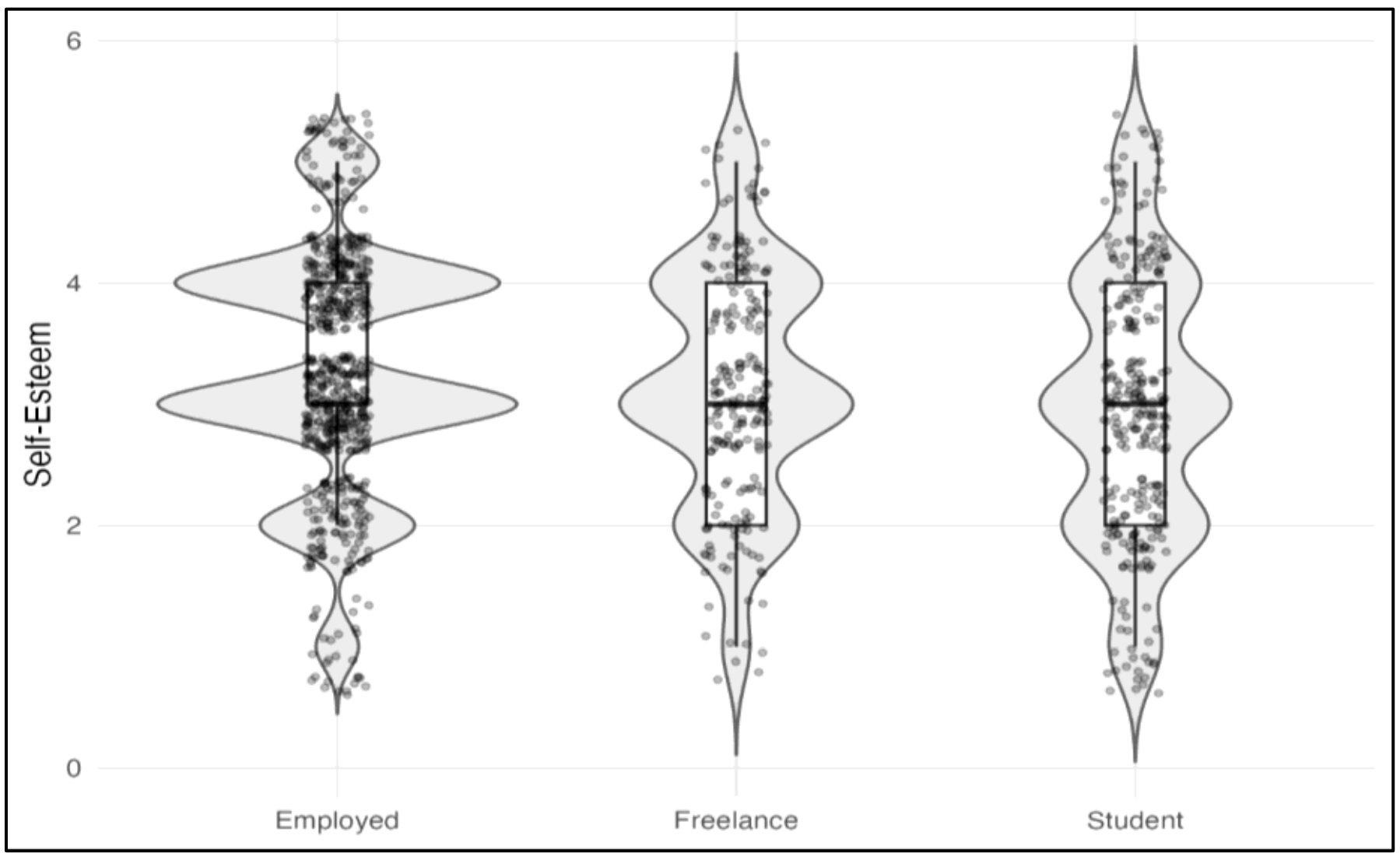

Figure S8. Distribution of depressive symptoms by gender at T1.

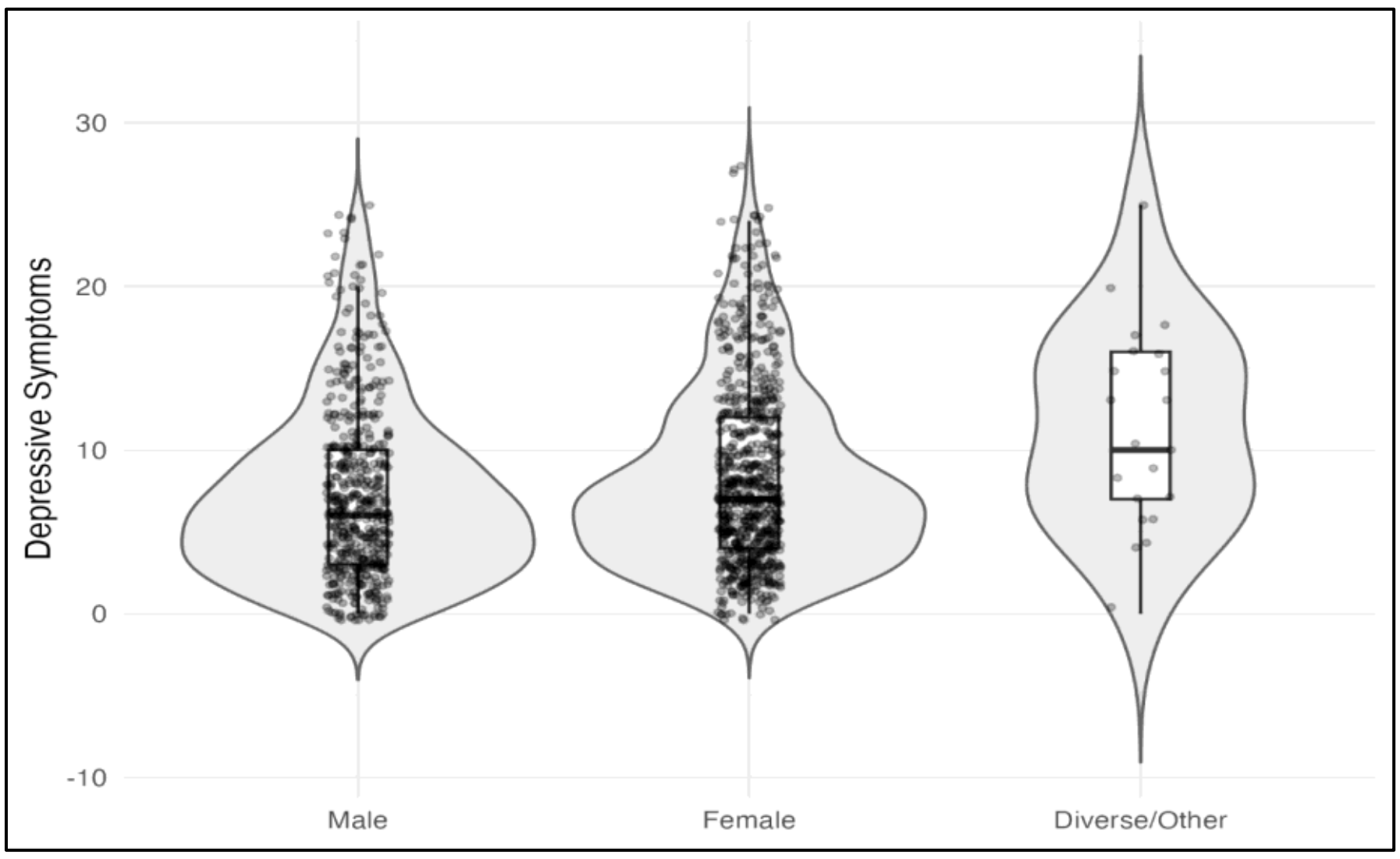

Figure S9. Distribution of self-efficacy by job role at T1.

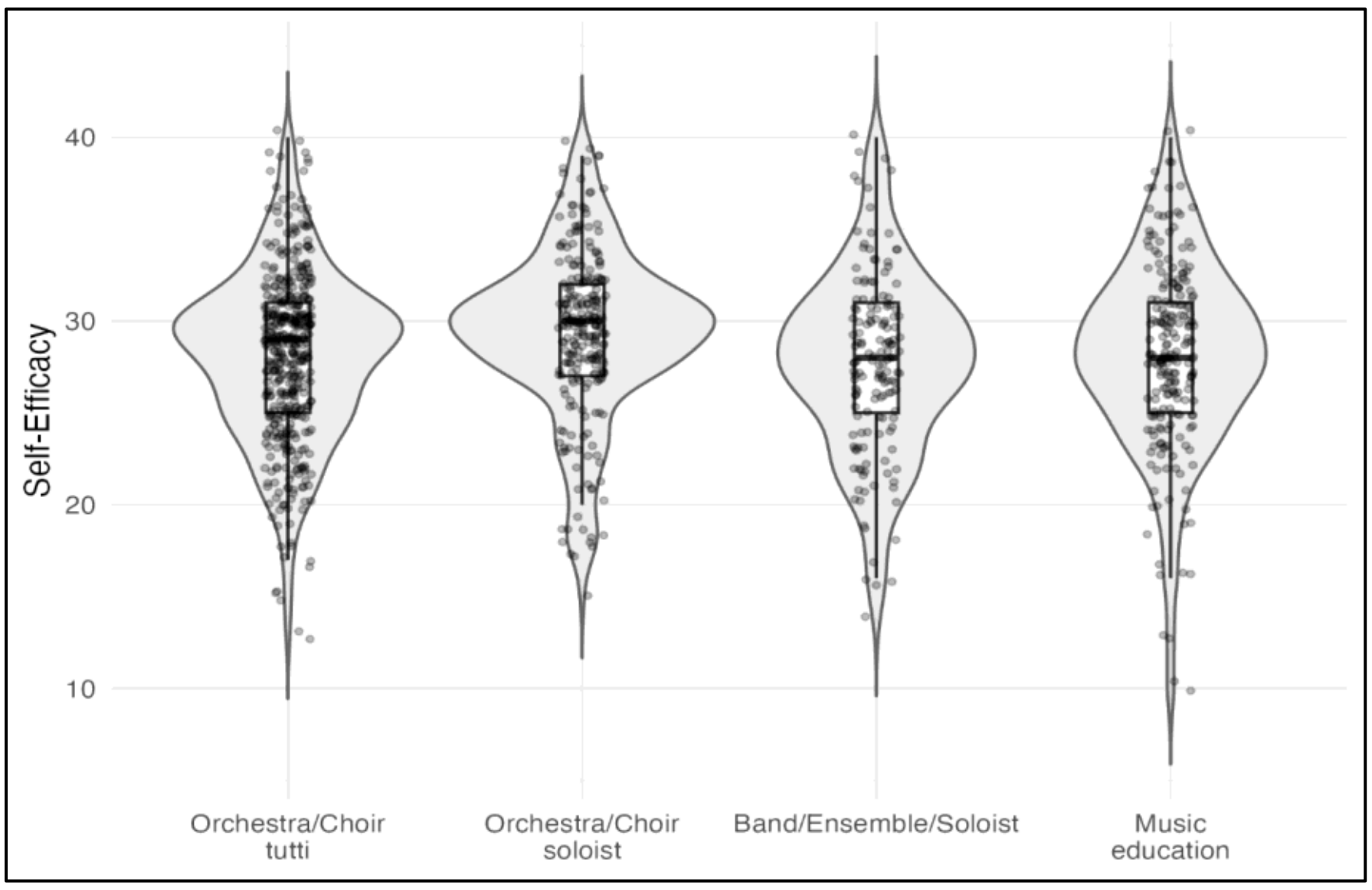

Figure S10. Distribution of self-efficacy by gender at T1.

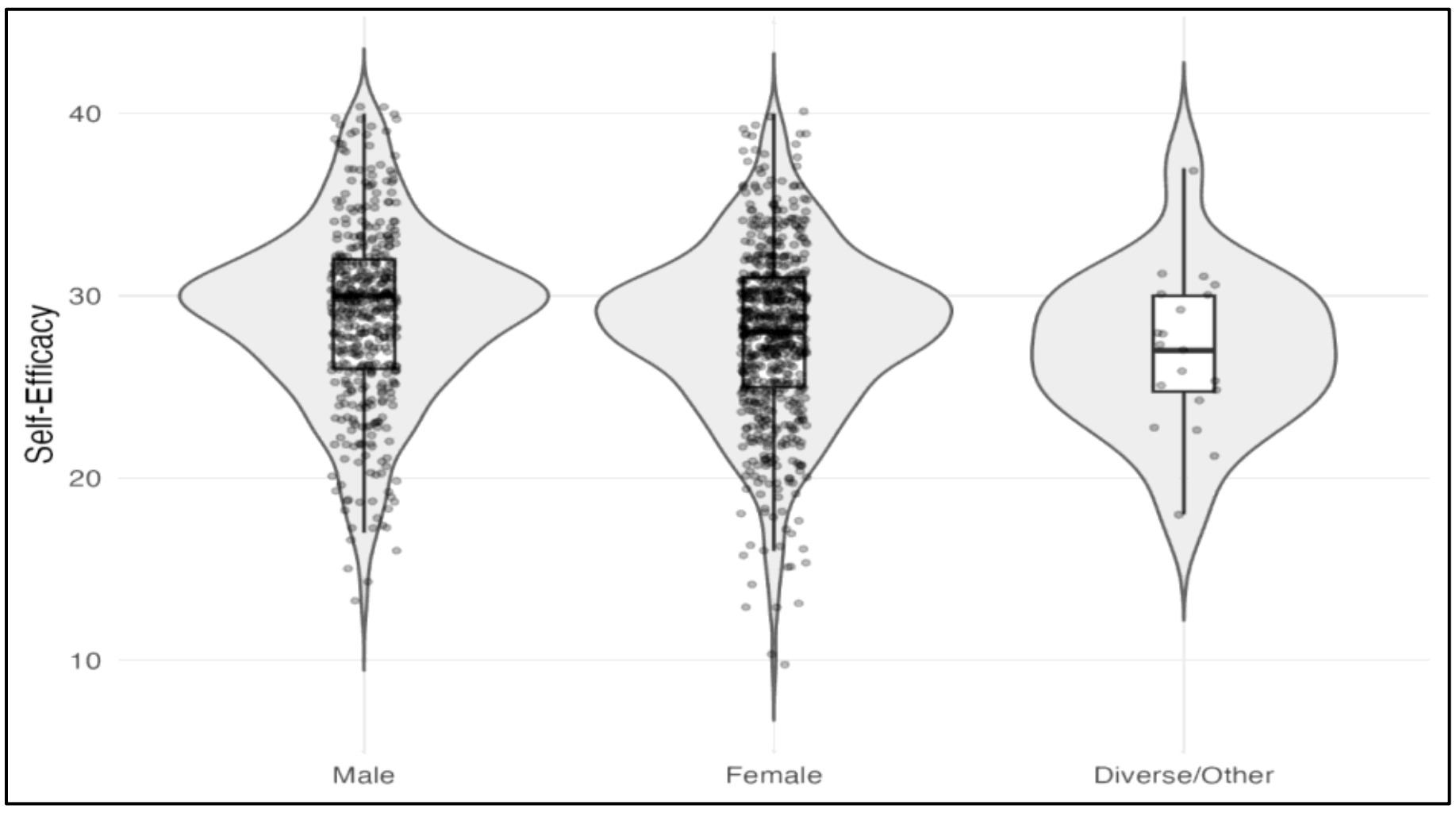

Figure S11. Distribution of self-efficacy by employment status at T1.

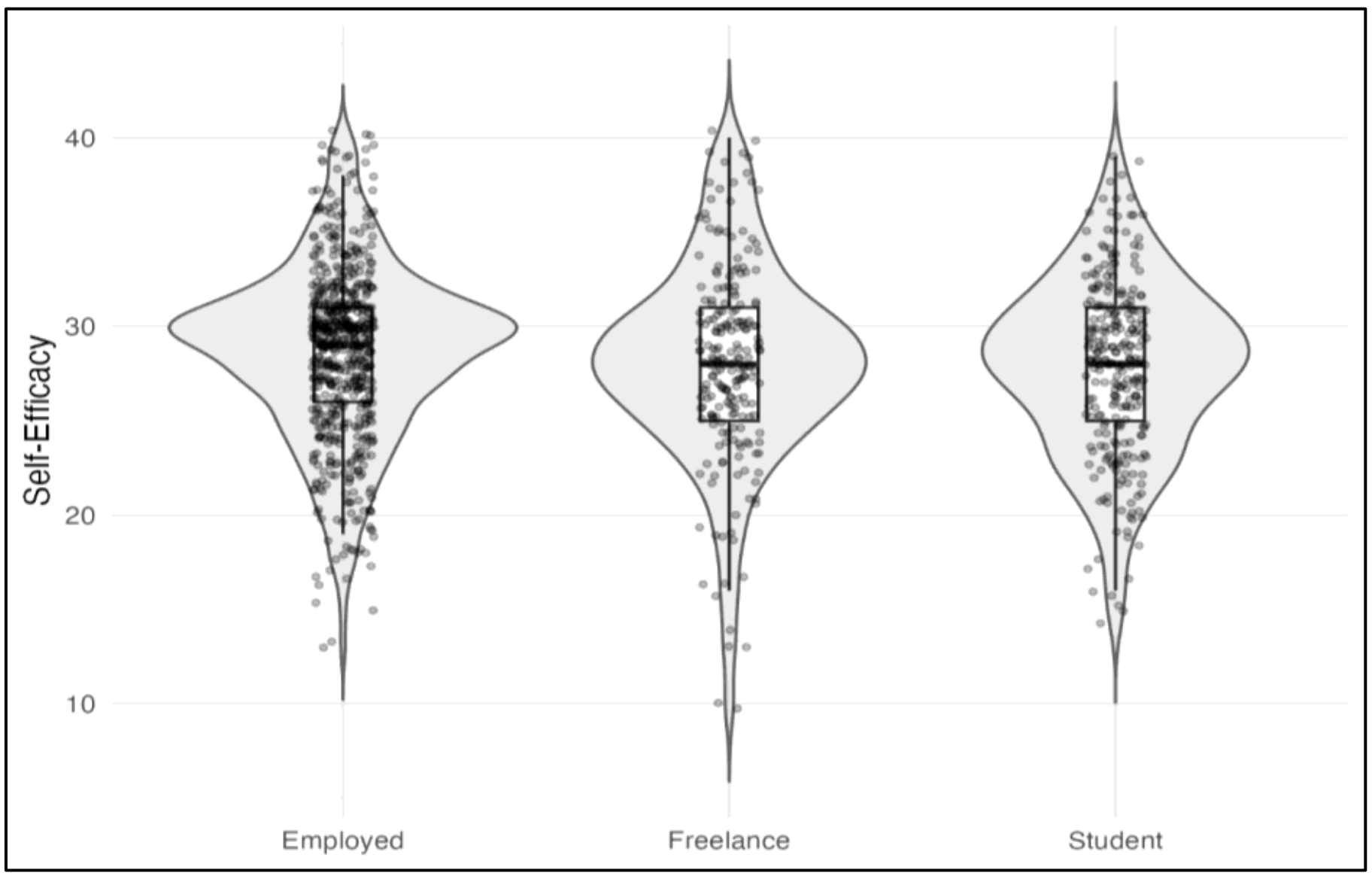

Figure S12. Distribution of resilience by employment status at T1.

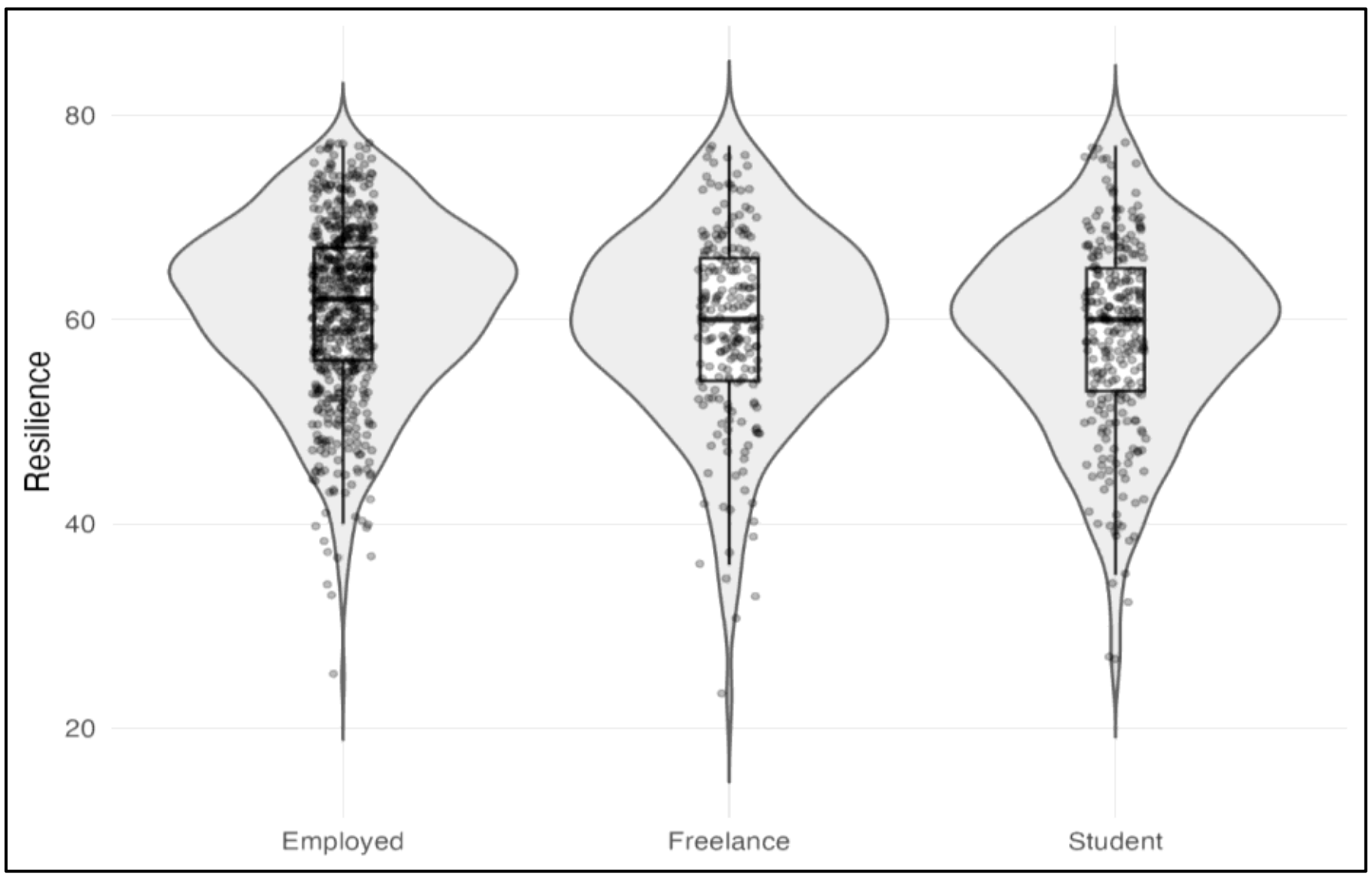

Supplement: Supplementary file 1 [file Table_1.pdf]
